# Supplementary figures and images for: Undercarboxylated osteocalcin inhibits the early differentiation of osteoclast mediated by Gprc6a
Source: PeerJ. 2021 Mar 2;9:e10898. doi: 10.7717/peerj.10898 (PMC7934677; doi:10.7717/peerj.10898)

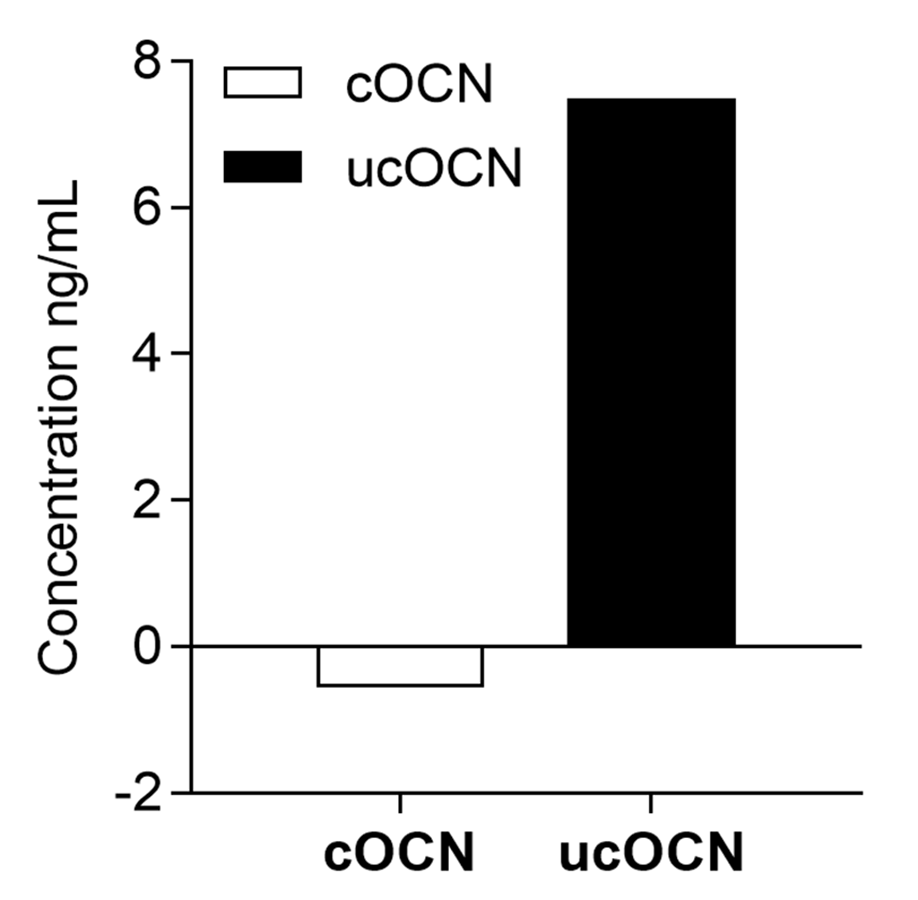

Supplement: Figure S1 — The purity and concentration of purified ucOCN from an artificial E.Coli prokaryotic bacteria by GST-fusion method was performed by ELISA using cOCN or ucOCN specific Kit. [file peerj-09-10898-s001.png]

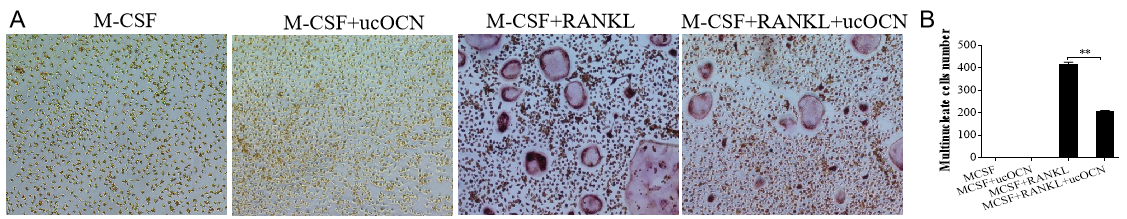

Supplement: Figure S2 — A: the TRAP staining of different treatment as above caption. B: the count of multinucleate cells and its analysis. [file peerj-09-10898-s002.png]

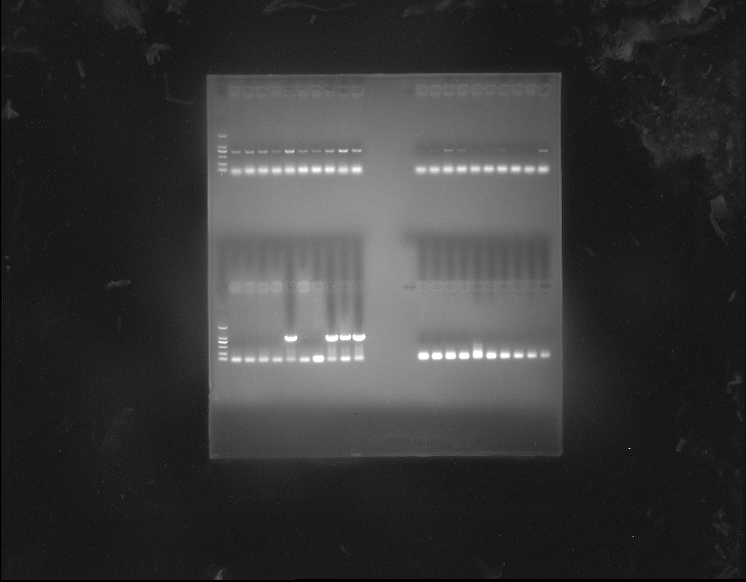

Supplement: Data S1 [file peerj-09-10898-s003.zip › 13116.TIF]

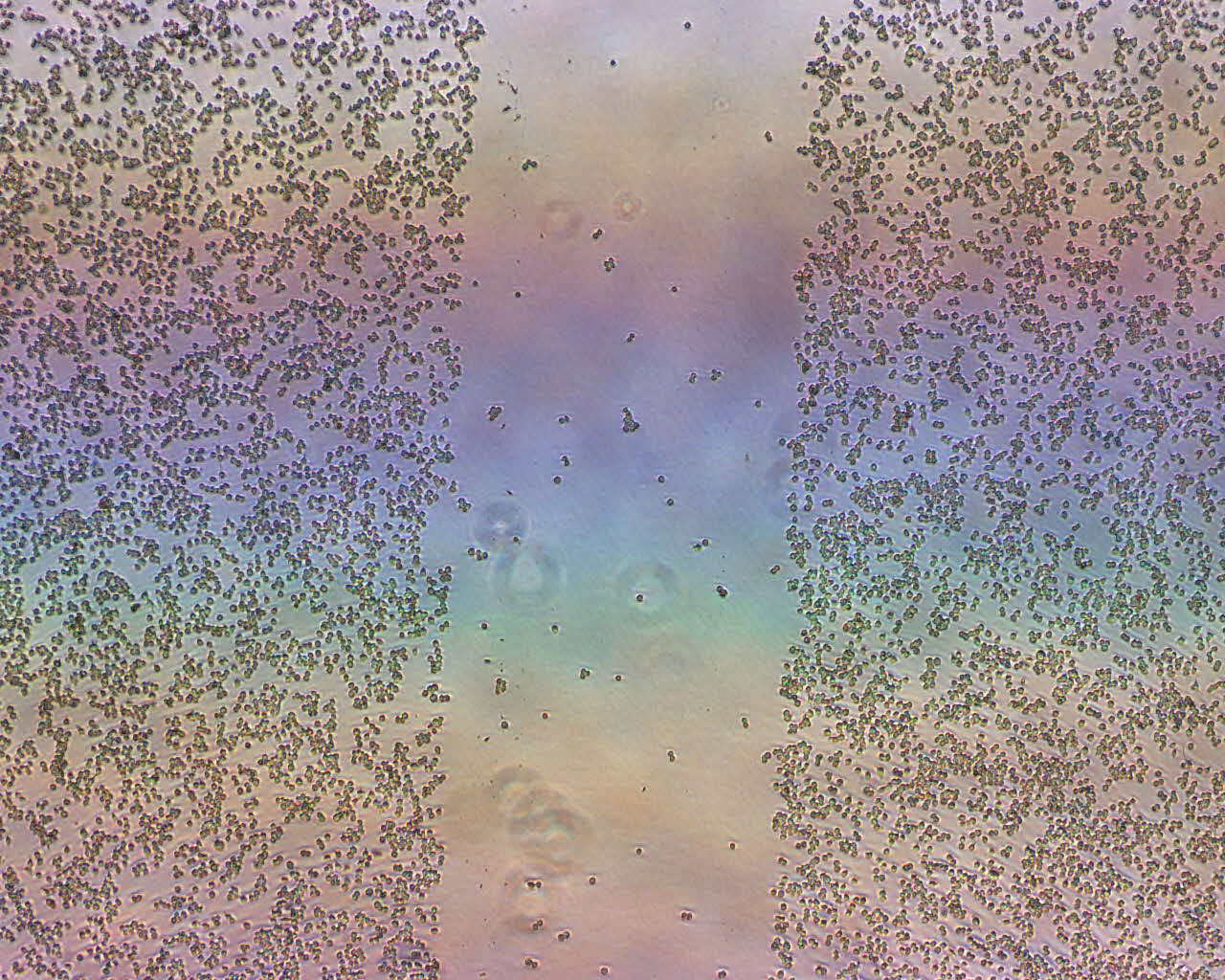

Supplement: Data S1 [file peerj-09-10898-s003.zip › Figure1B CN0h.jpg]

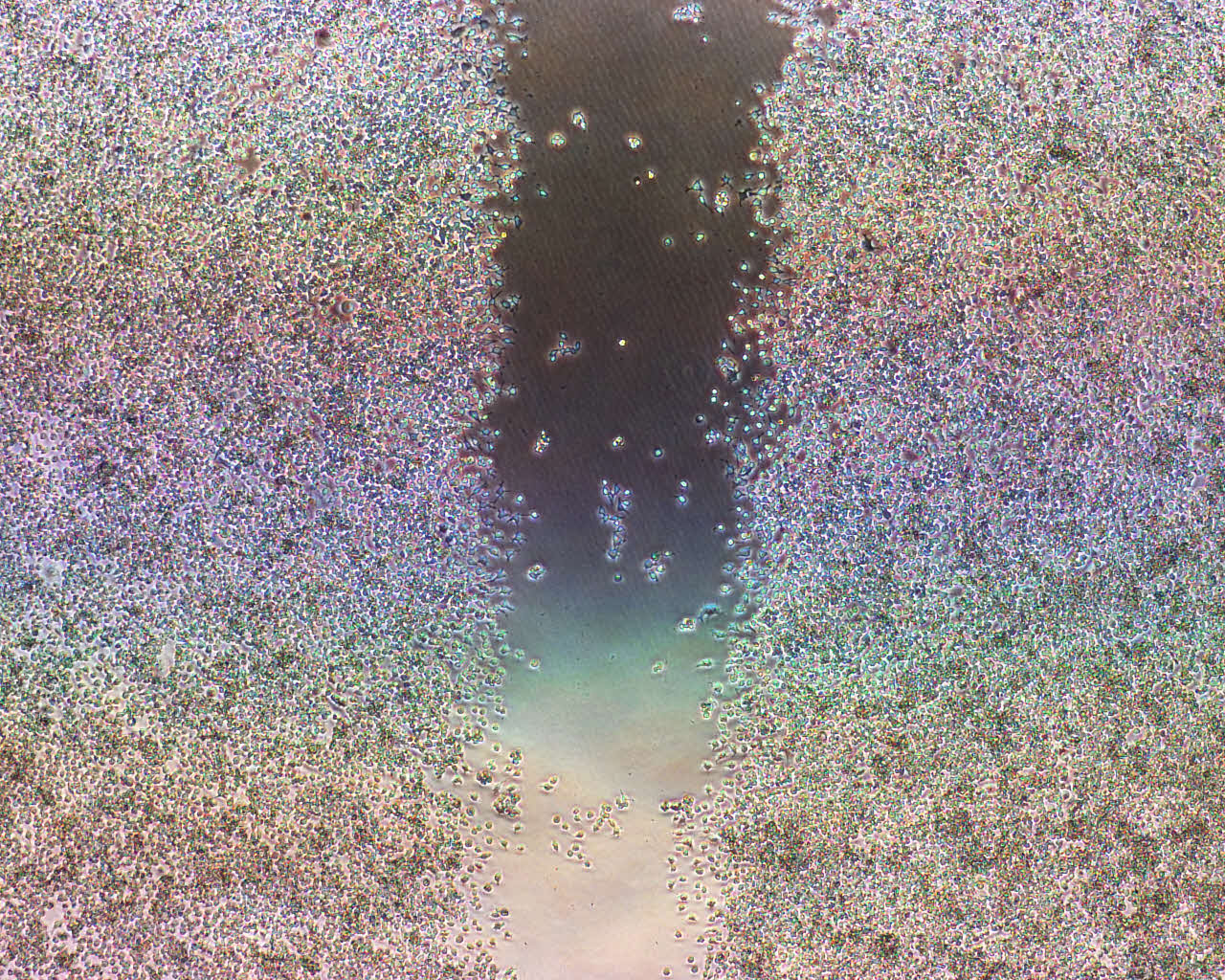

Supplement: Data S1 [file peerj-09-10898-s003.zip › Figure1B CN24h.jpg]

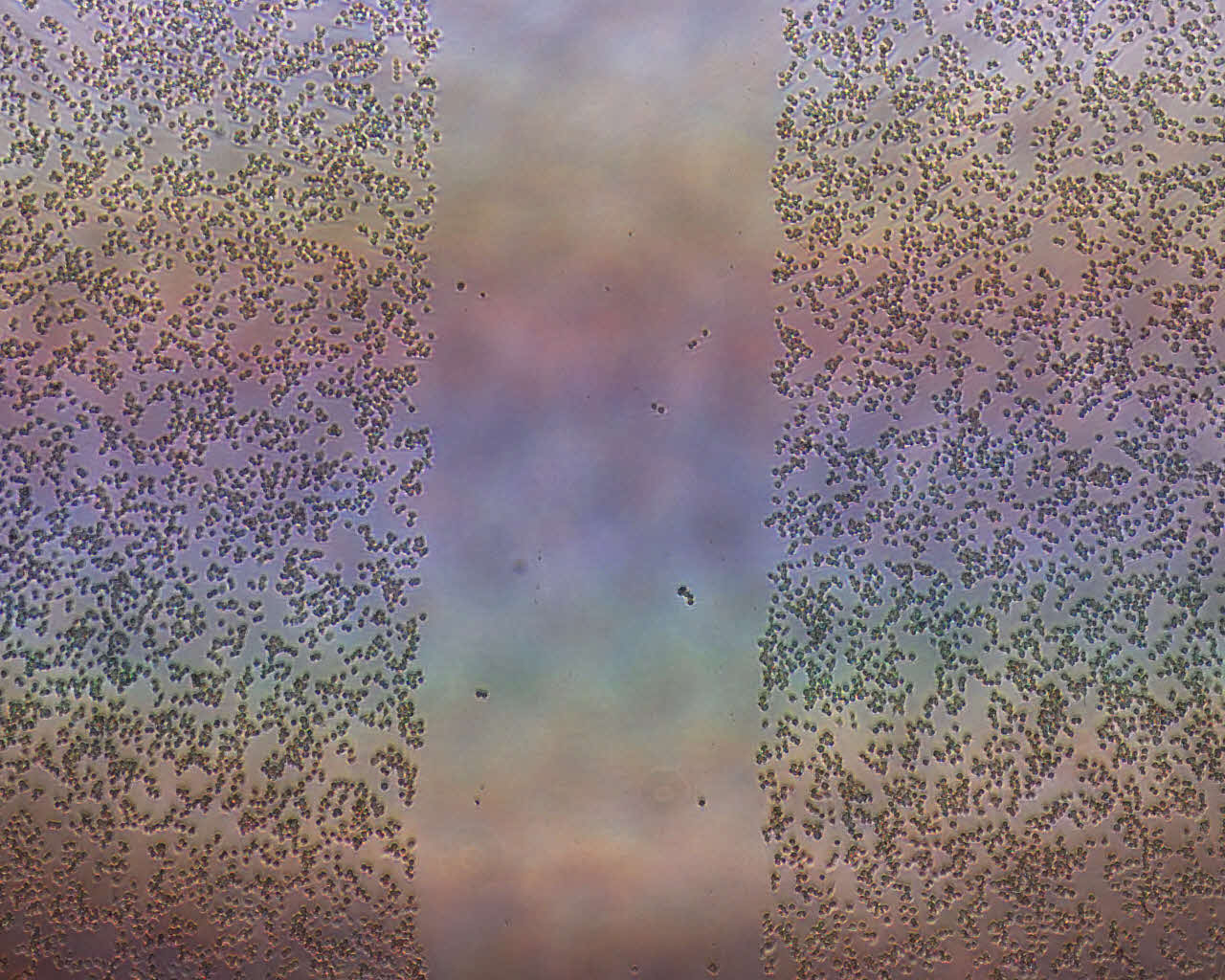

Supplement: Data S1 [file peerj-09-10898-s003.zip › Figure1B ucOCN0h.jpg]

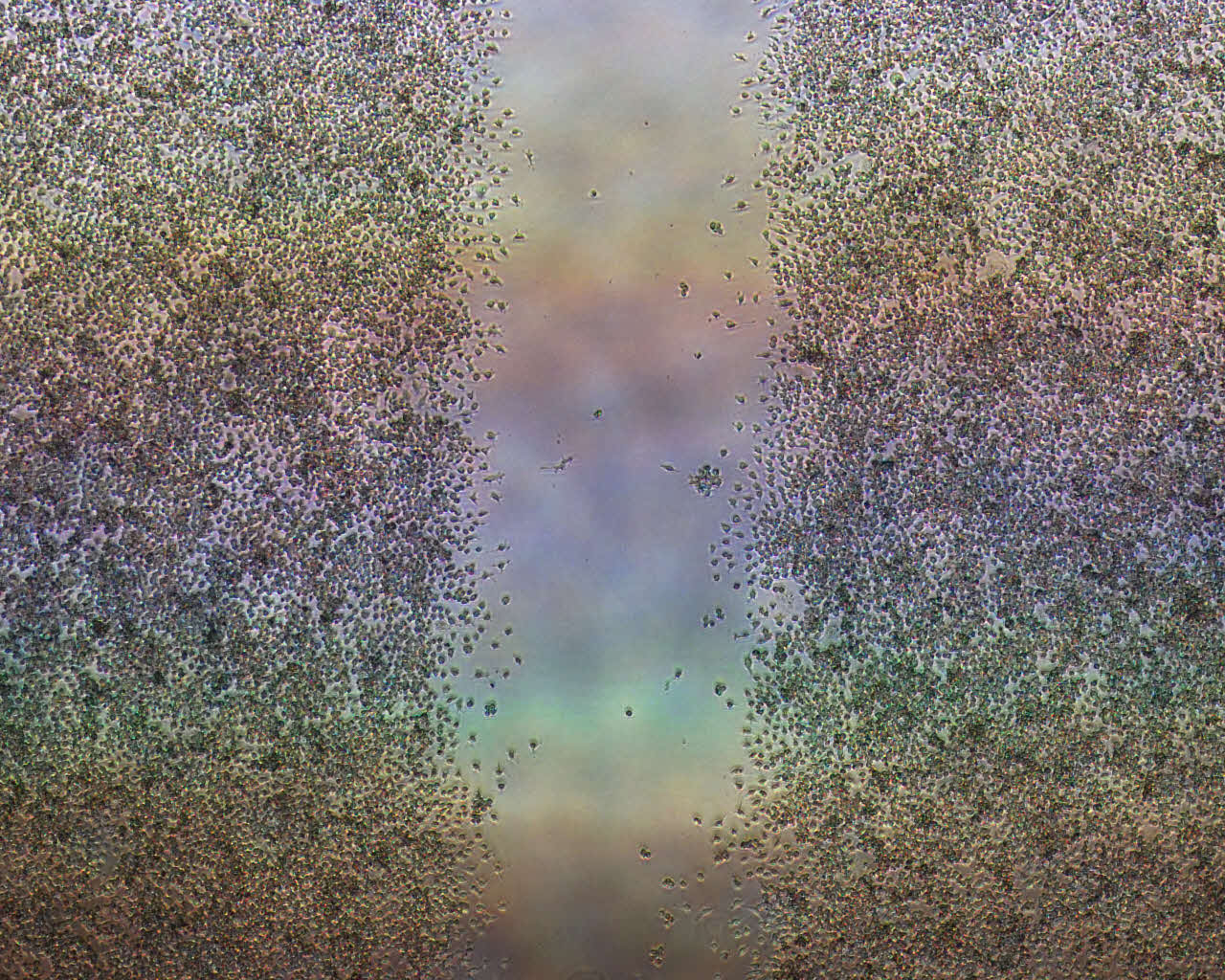

Supplement: Data S1 [file peerj-09-10898-s003.zip › Figure1B ucOCN24h.jpg]

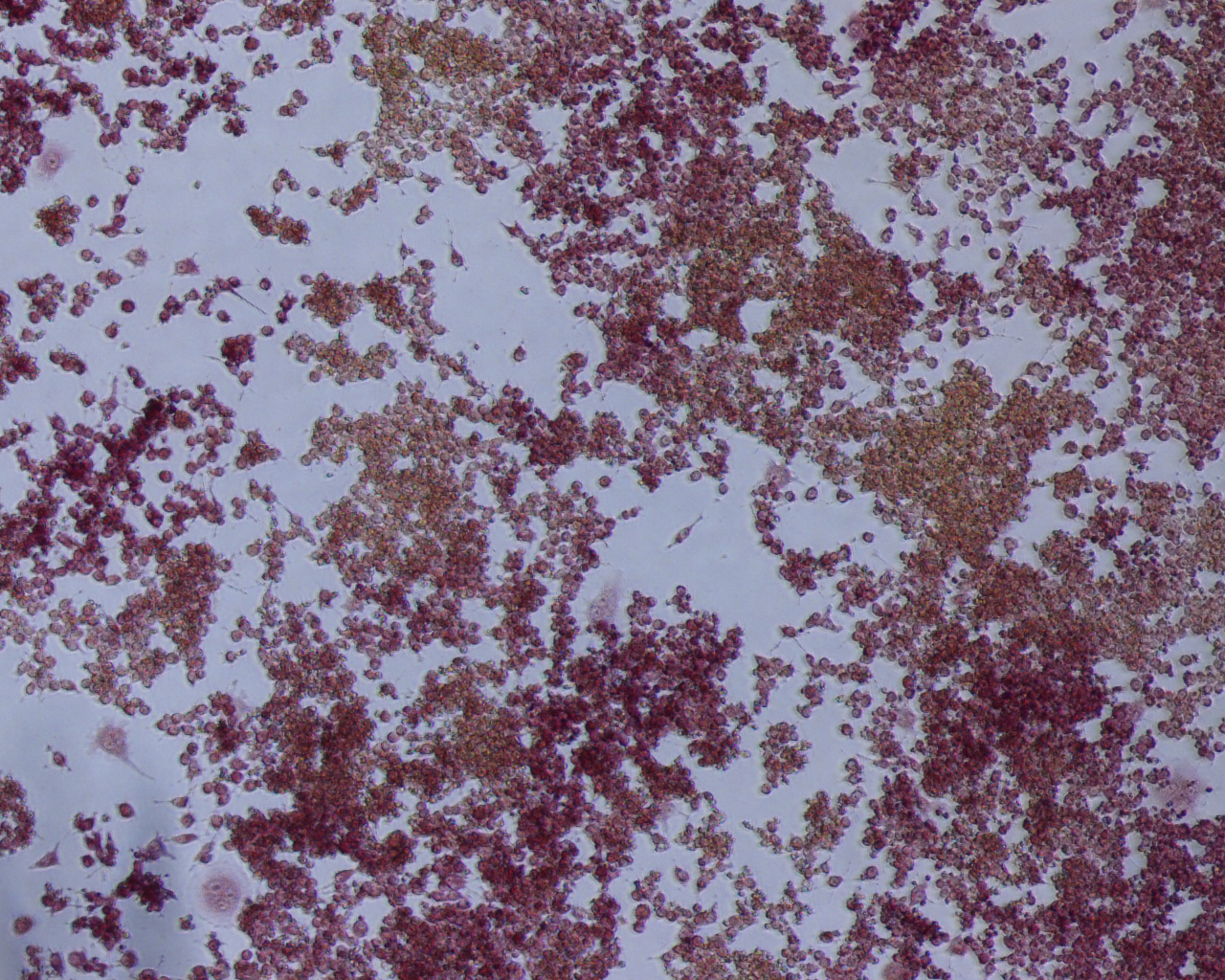

Supplement: Data S1 [file peerj-09-10898-s003.zip › Figure3A-Medium+Rankl-ucOCN.jpg]

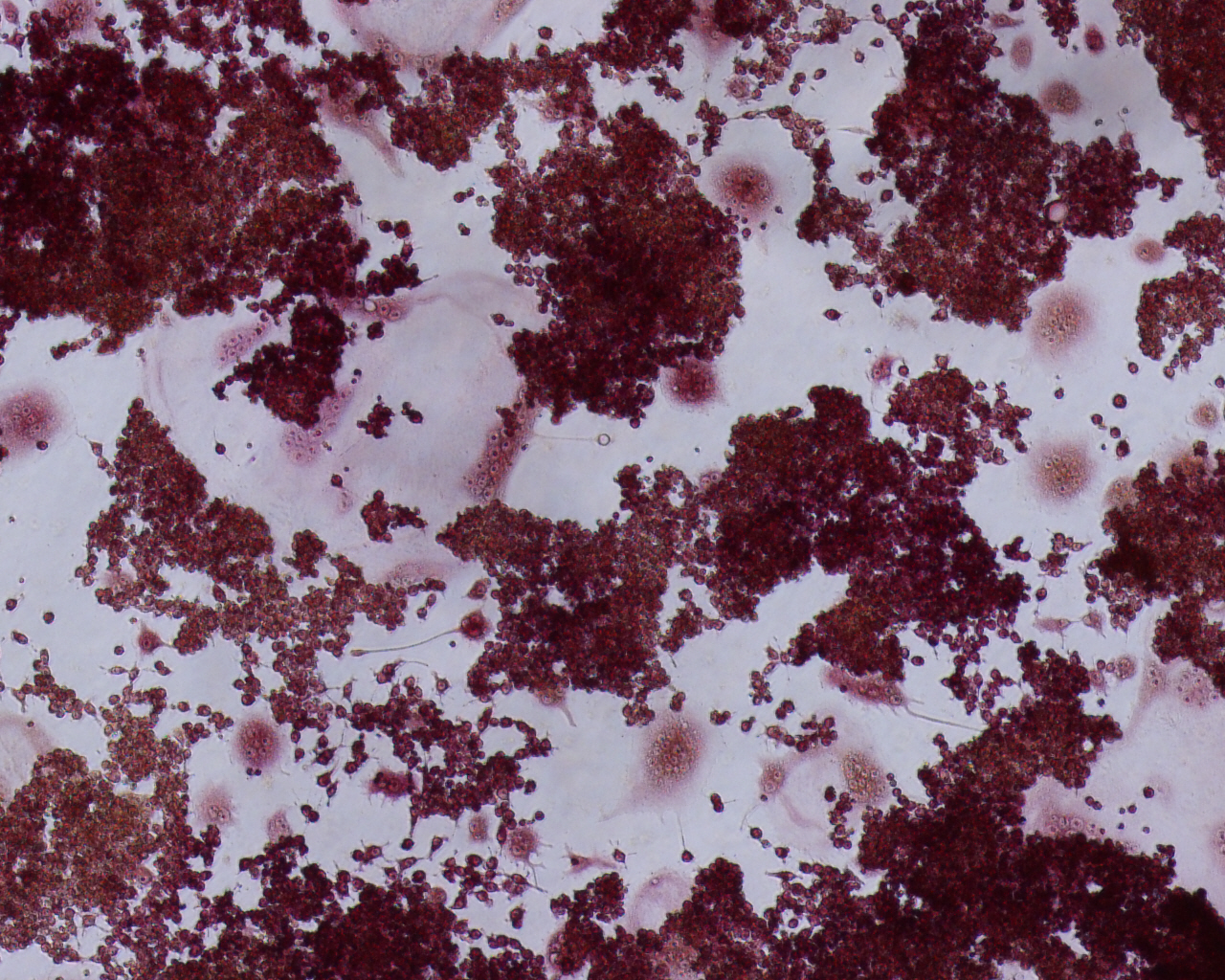

Supplement: Data S1 [file peerj-09-10898-s003.zip › Figure3A-Medium+RANKL.jpg]

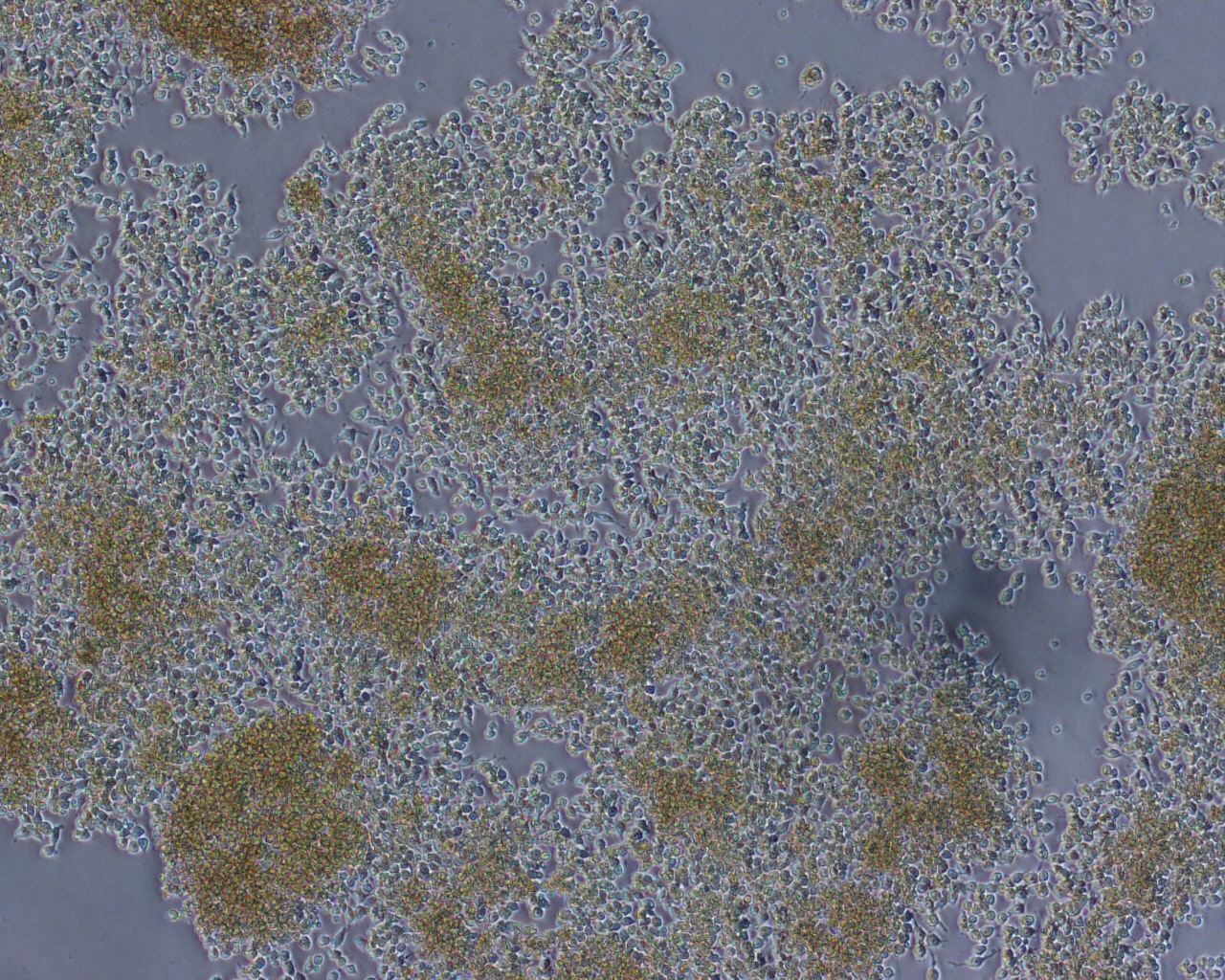

Supplement: Data S1 [file peerj-09-10898-s003.zip › Figure3A-Medium.jpg]

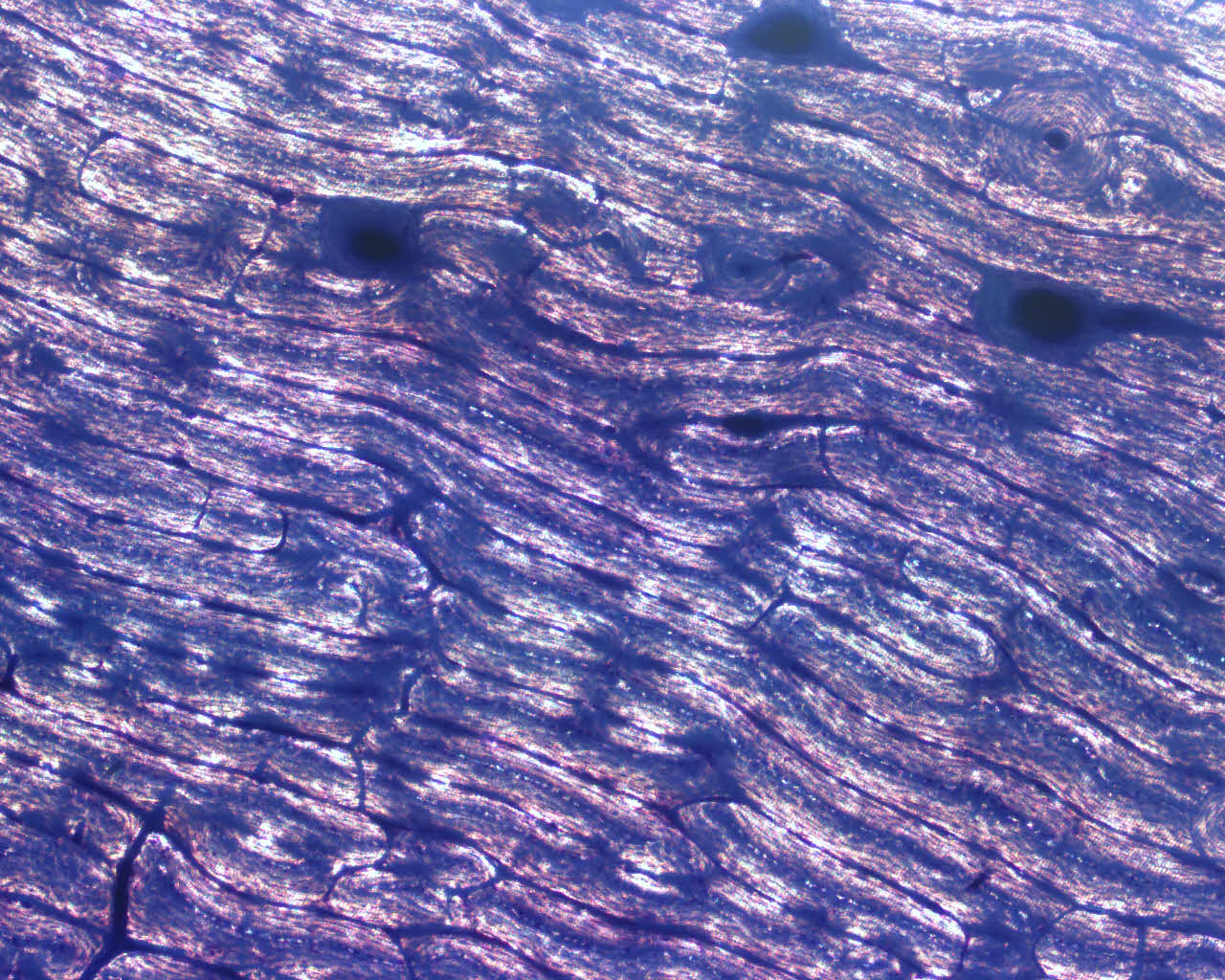

Supplement: Data S1 [file peerj-09-10898-s003.zip › Figure3B-medium+Rankl+ucOCN.jpg]

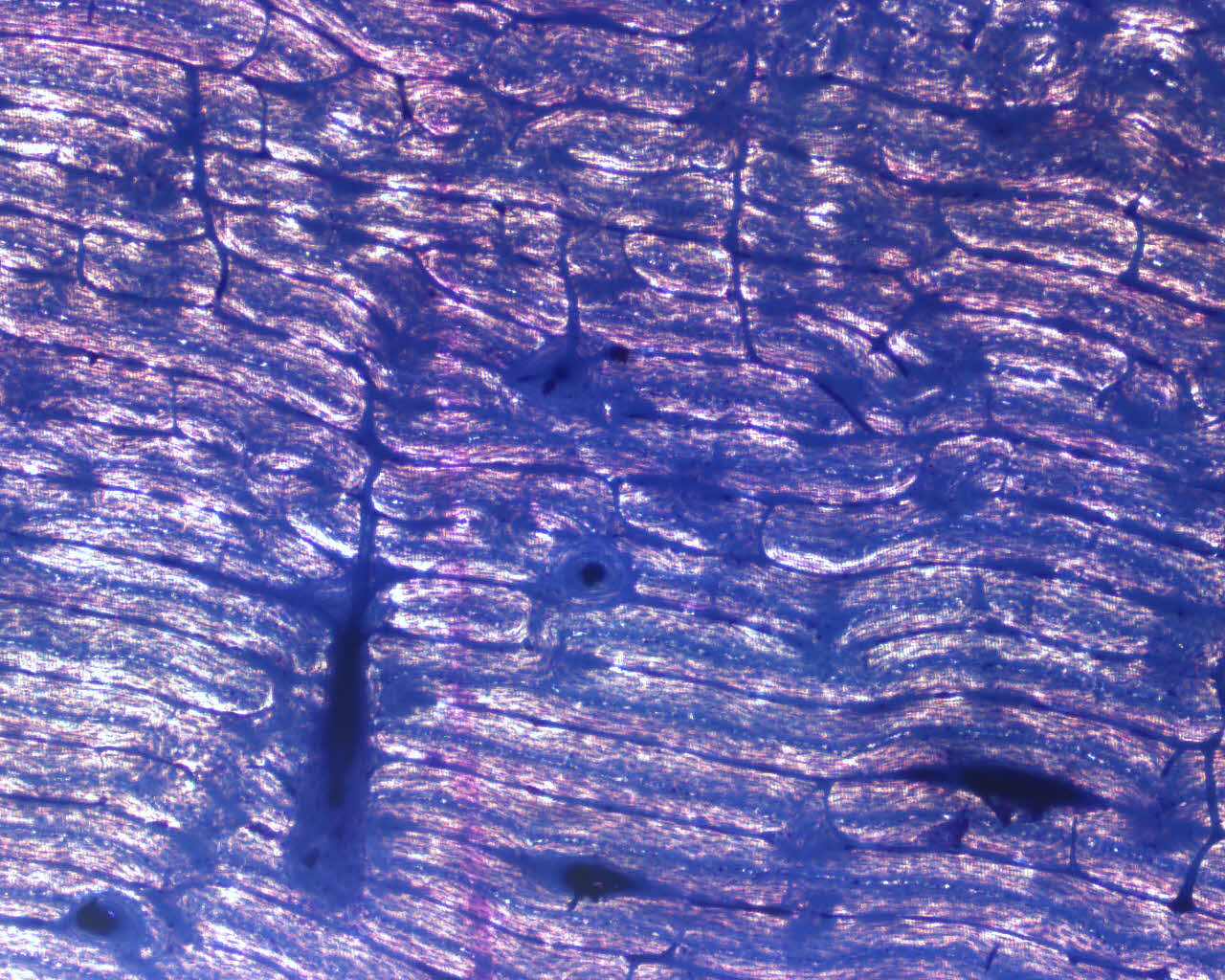

Supplement: Data S1 [file peerj-09-10898-s003.zip › Figure3B-medium+Rankl.jpg]

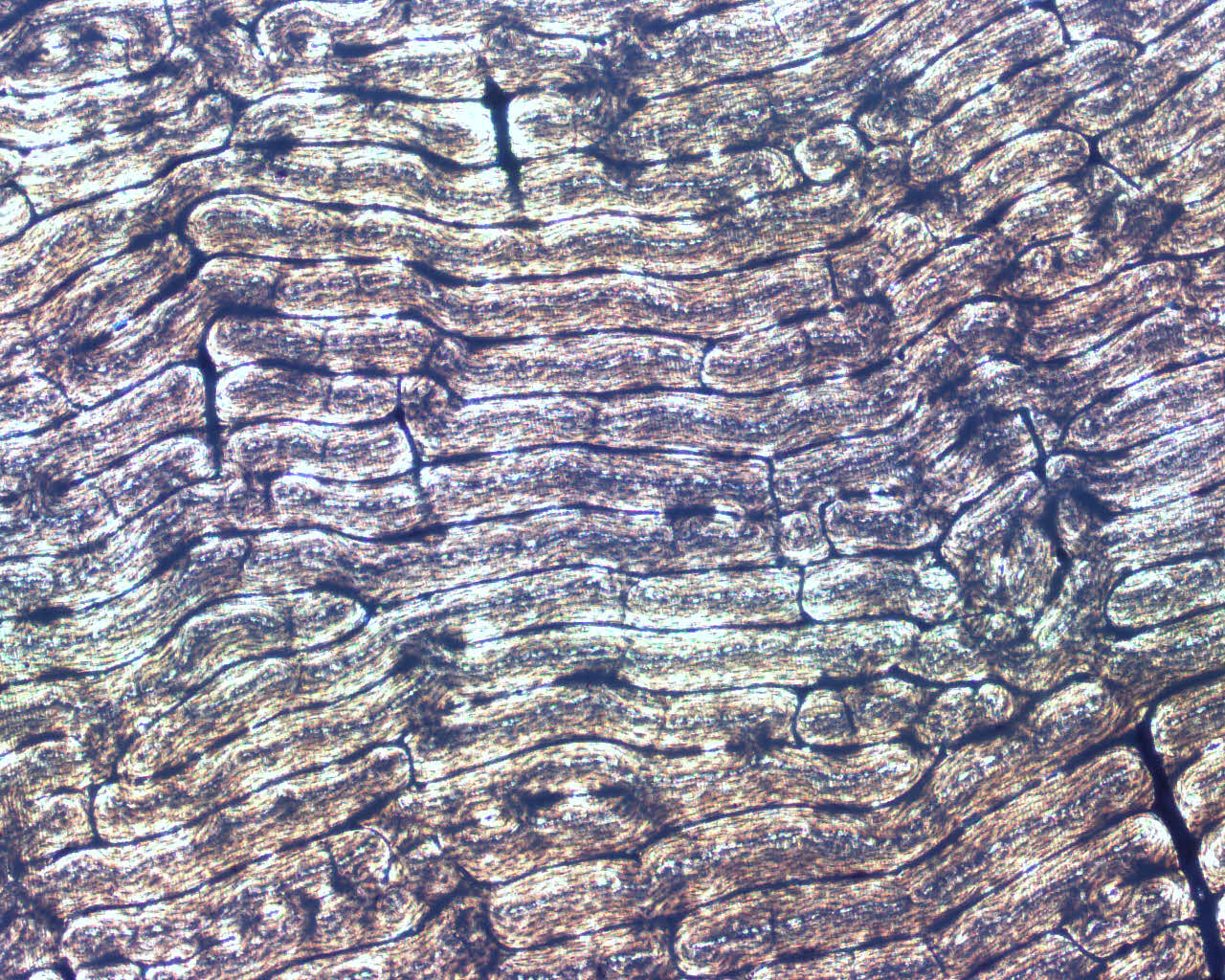

Supplement: Data S1 [file peerj-09-10898-s003.zip › Figure3B-medium.jpg]

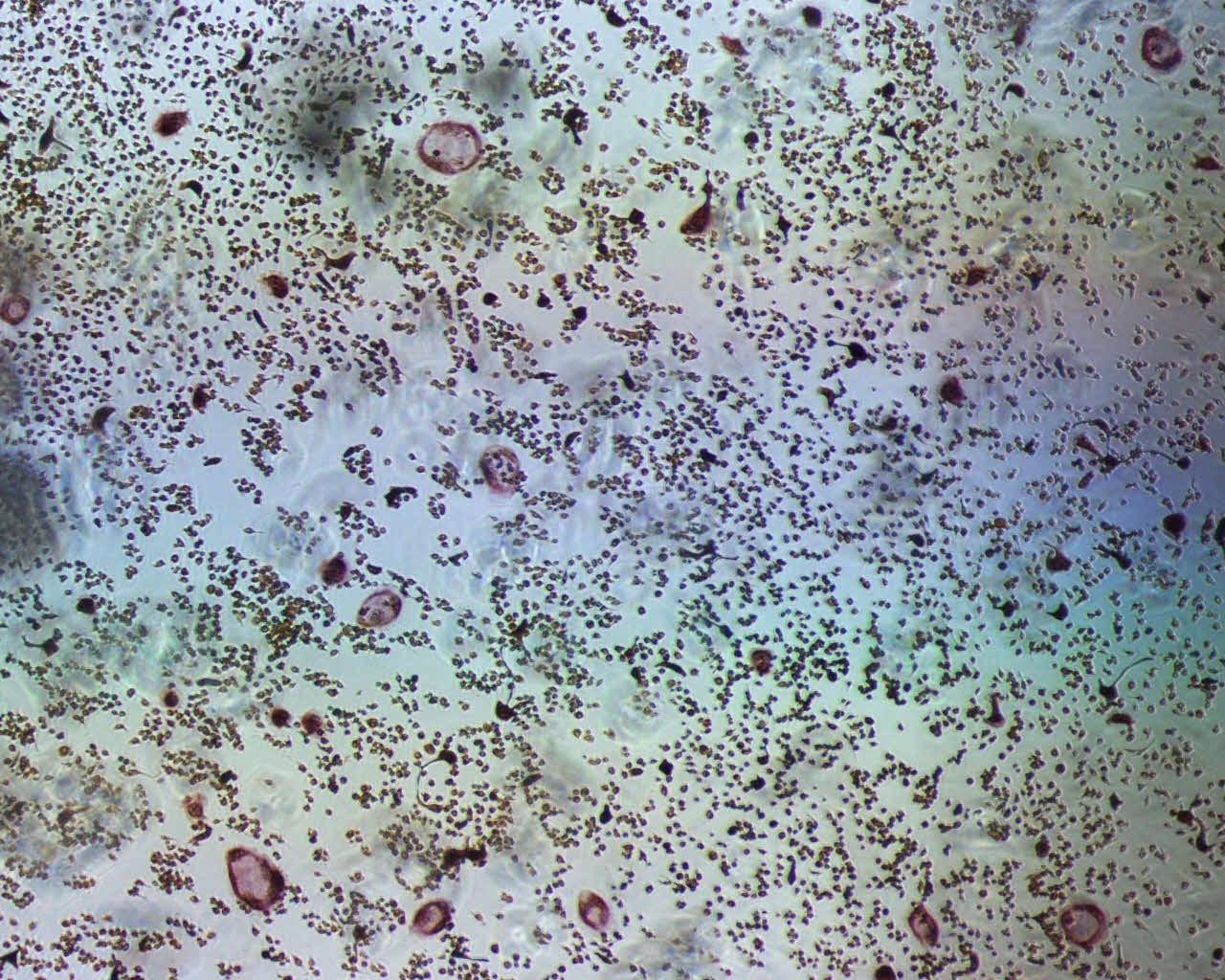

Supplement: Data S1 [file peerj-09-10898-s003.zip › Figure3C-MCSF+RANKL+ucOCN.jpg]

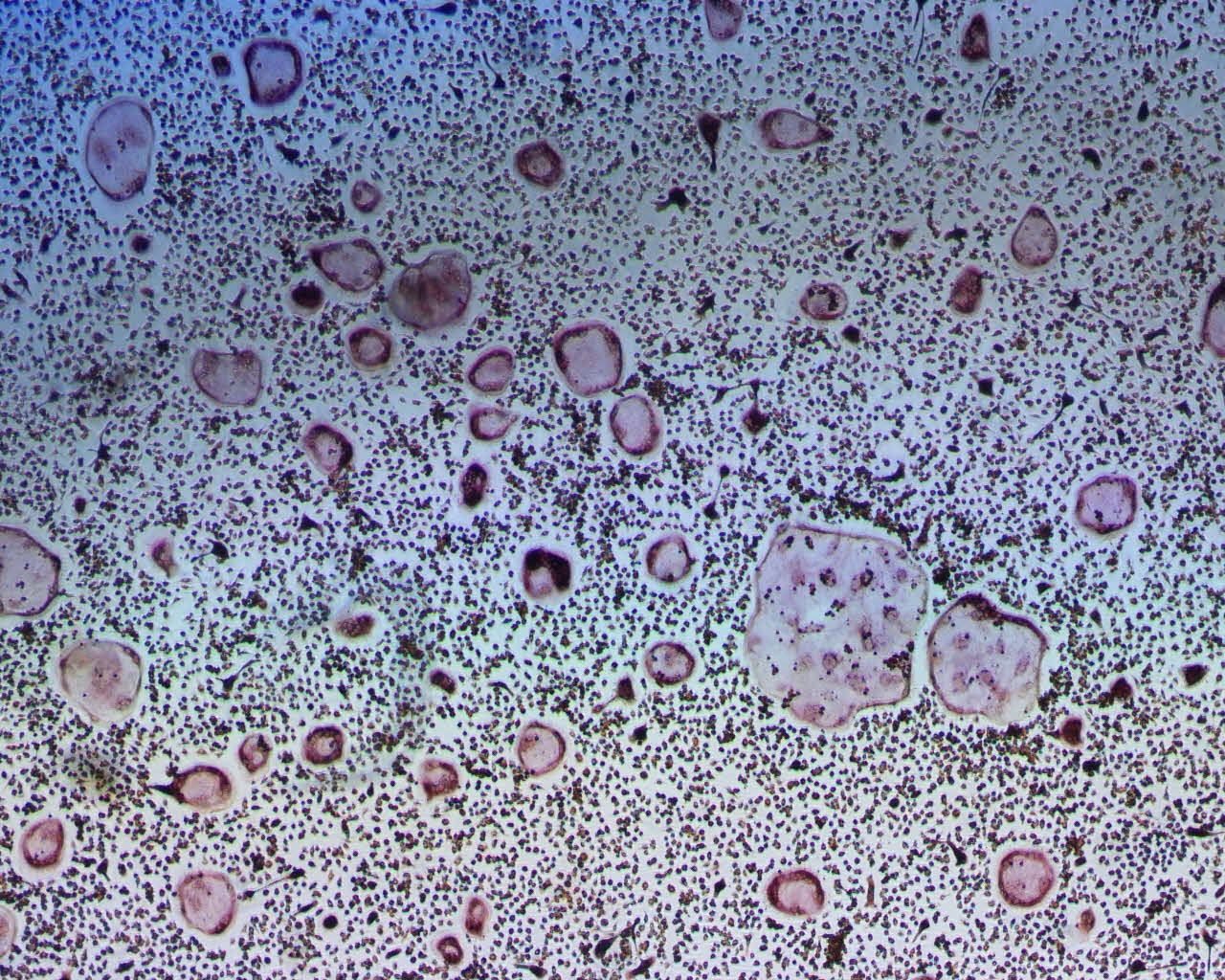

Supplement: Data S1 [file peerj-09-10898-s003.zip › Figure3C-MCSF+RANKL.jpg]

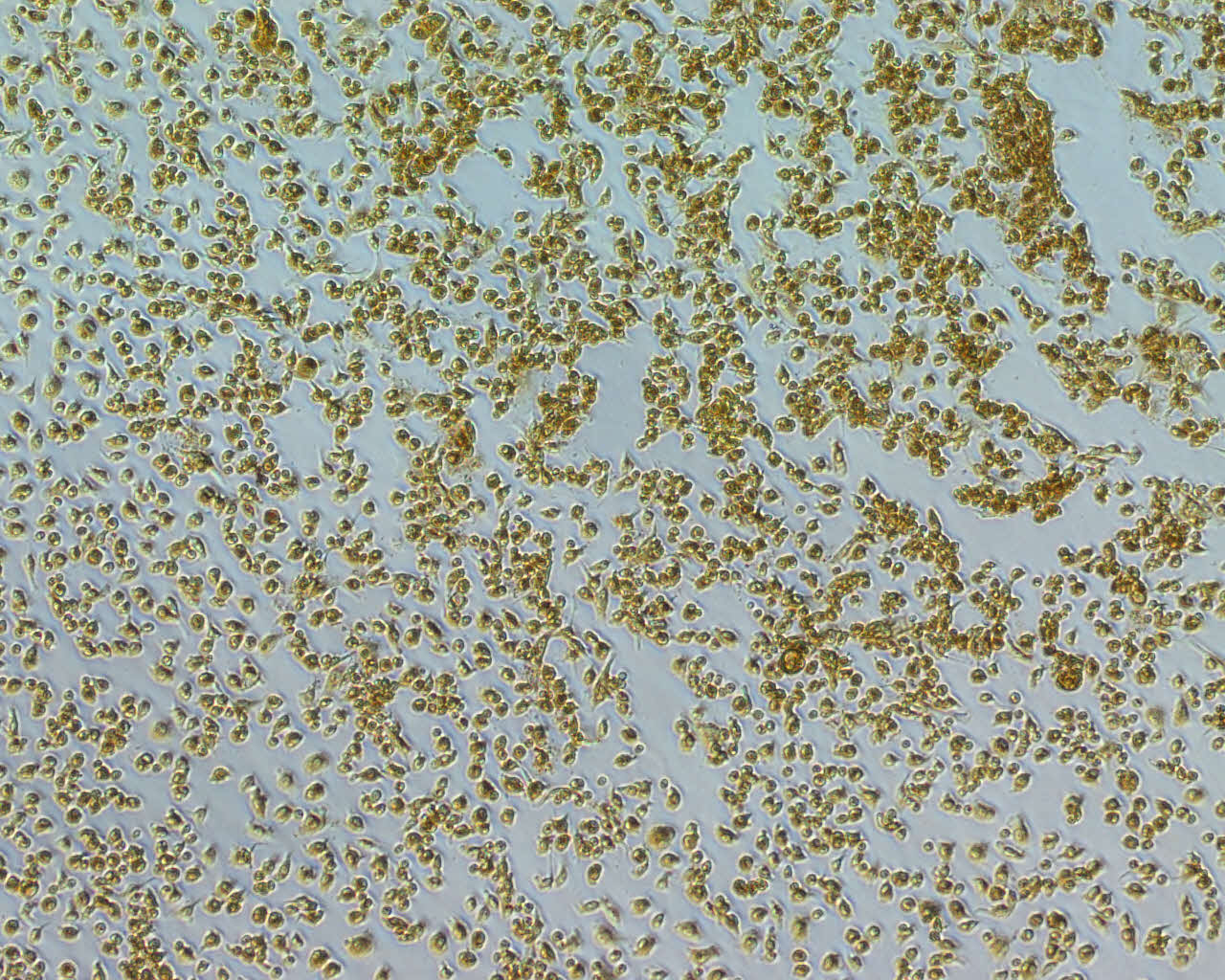

Supplement: Data S1 [file peerj-09-10898-s003.zip › Figure3C-MCSF.jpg]

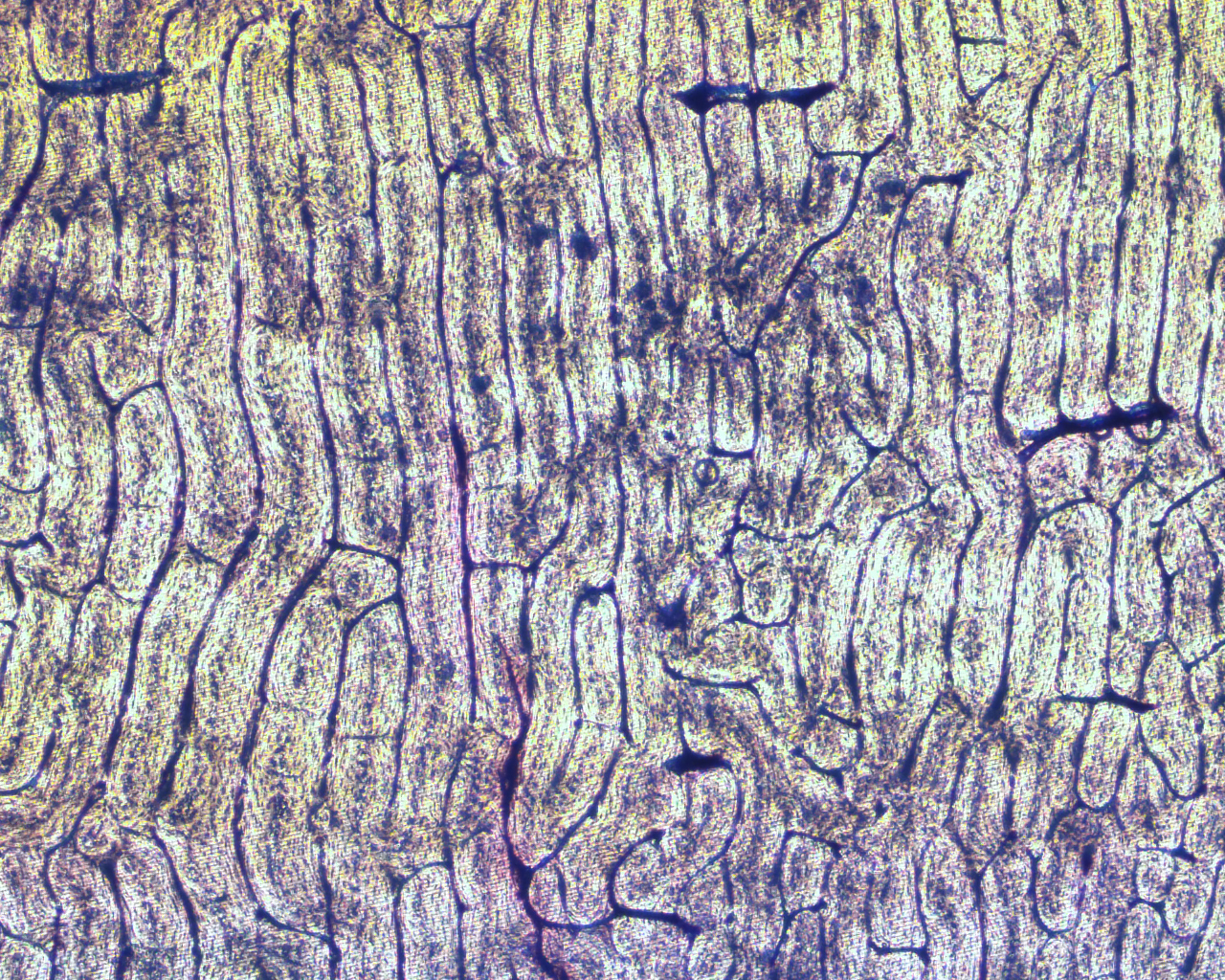

Supplement: Data S1 [file peerj-09-10898-s003.zip › Figure3D-MCSF+RANKL+ucOCN.jpg]

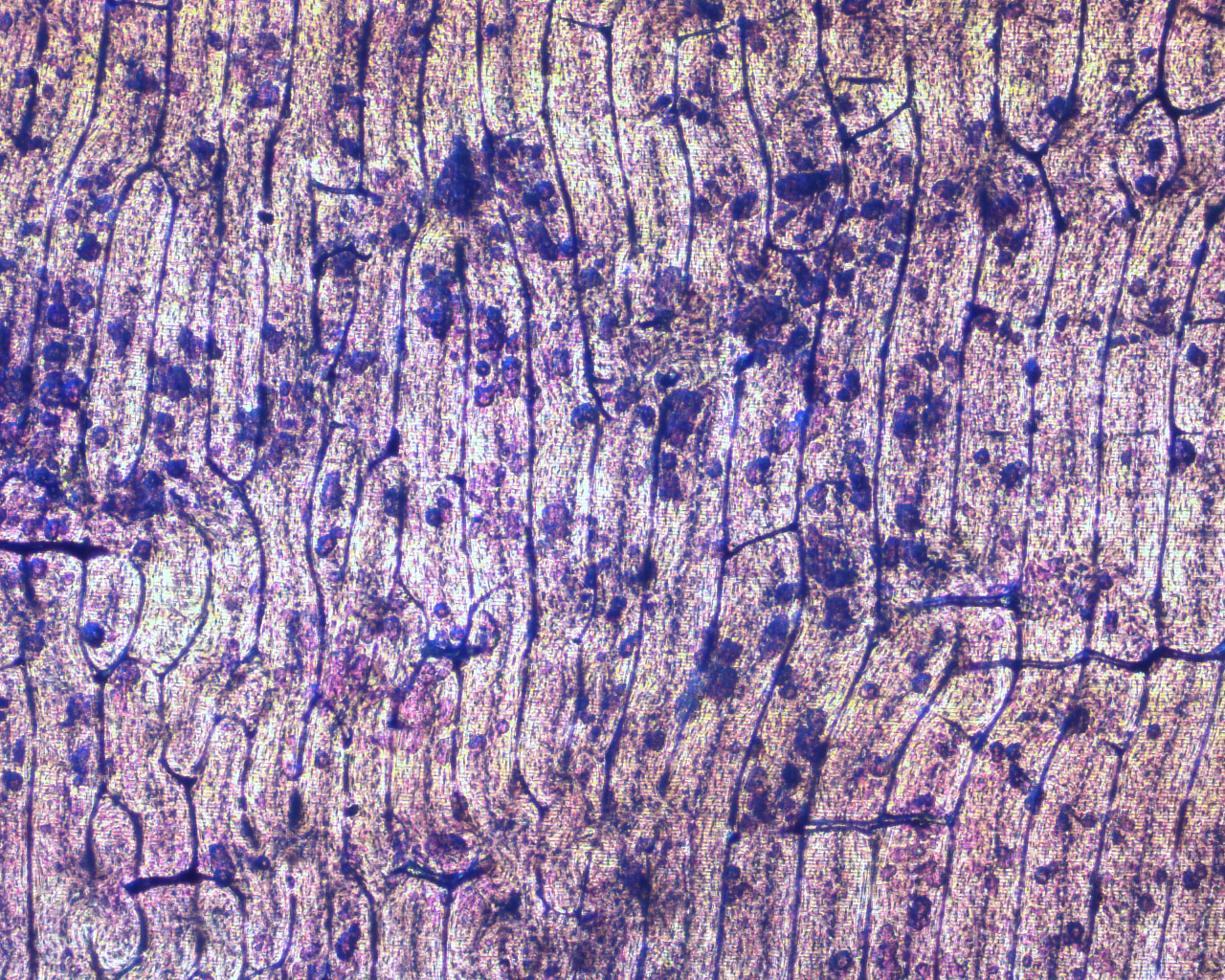

Supplement: Data S1 [file peerj-09-10898-s003.zip › Figure3D-MCSF+RANKL.jpg]

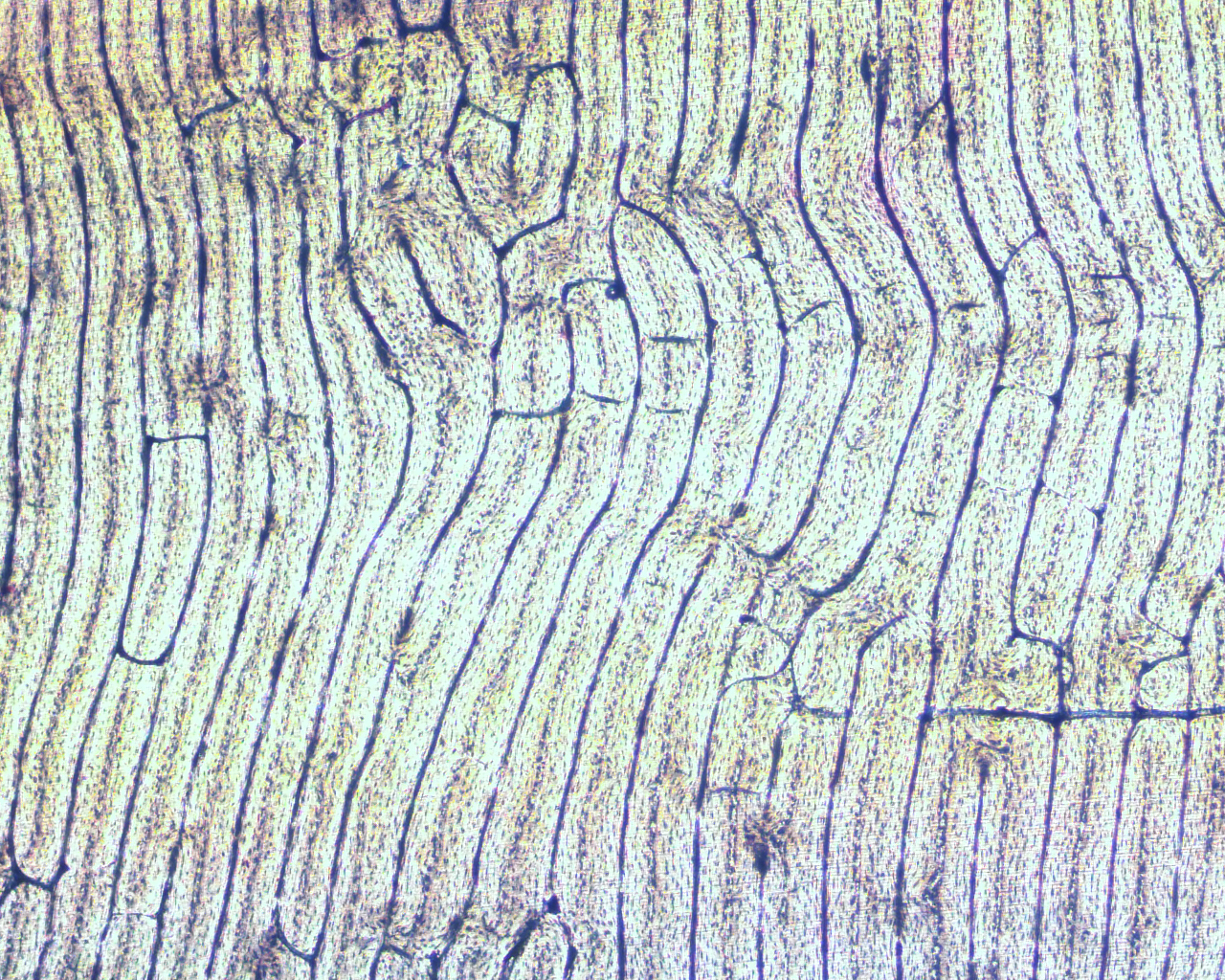

Supplement: Data S1 [file peerj-09-10898-s003.zip › Figure3D-MCSF.jpg]

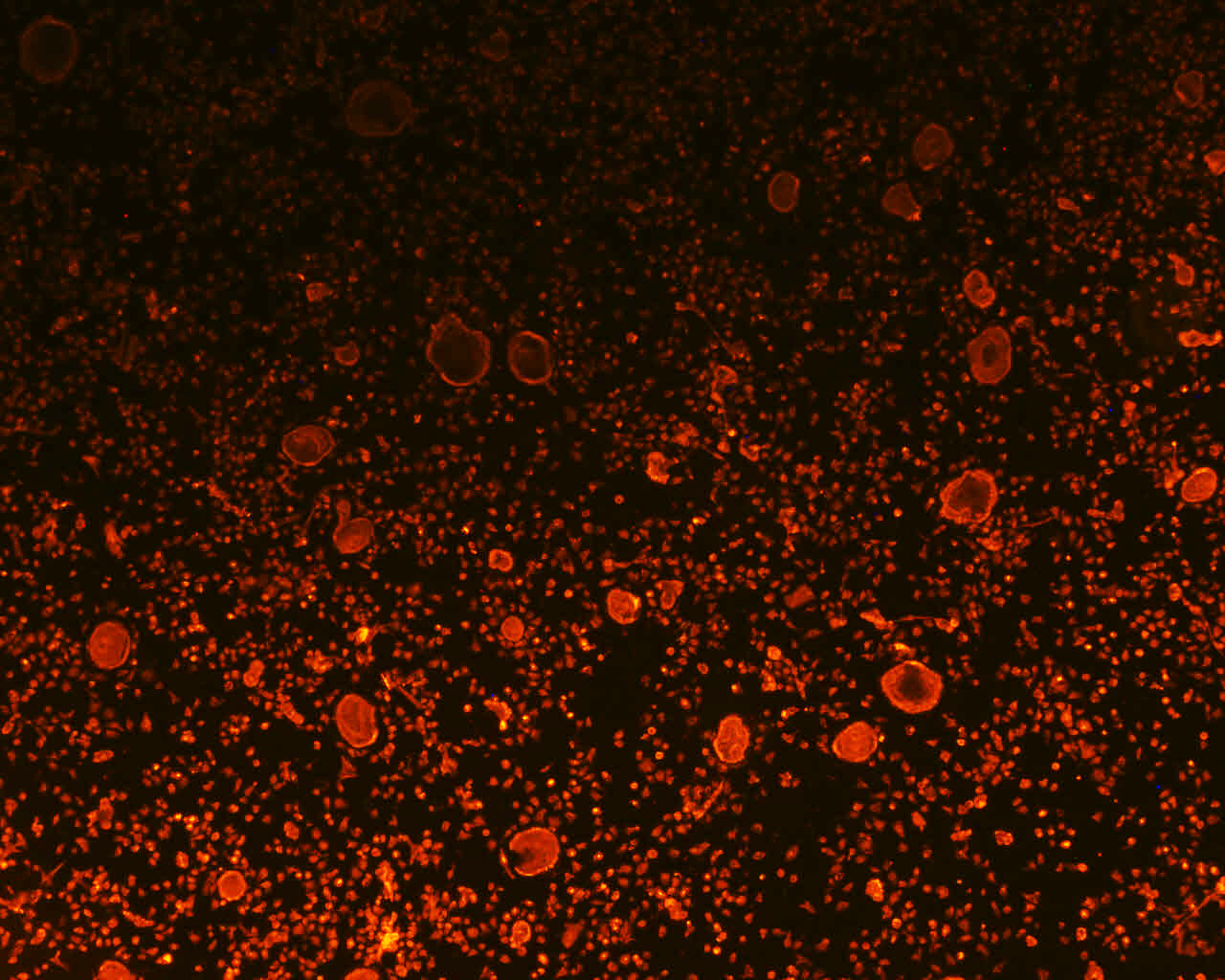

Supplement: Data S1 [file peerj-09-10898-s003.zip › Figure3E-MCSF+RANKL+ucOCN.tif]

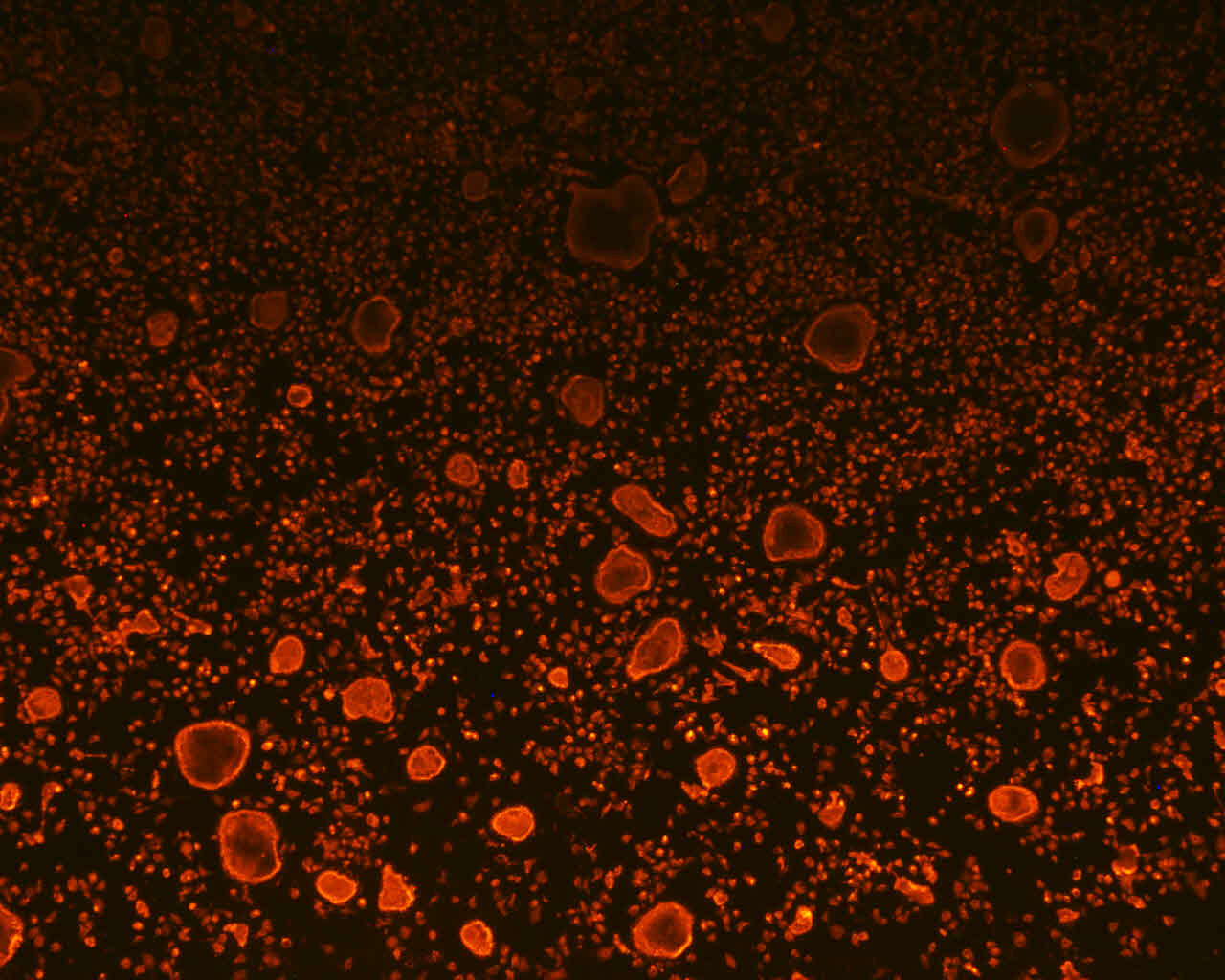

Supplement: Data S1 [file peerj-09-10898-s003.zip › Figure3E-MCSF+RANKL.tif]

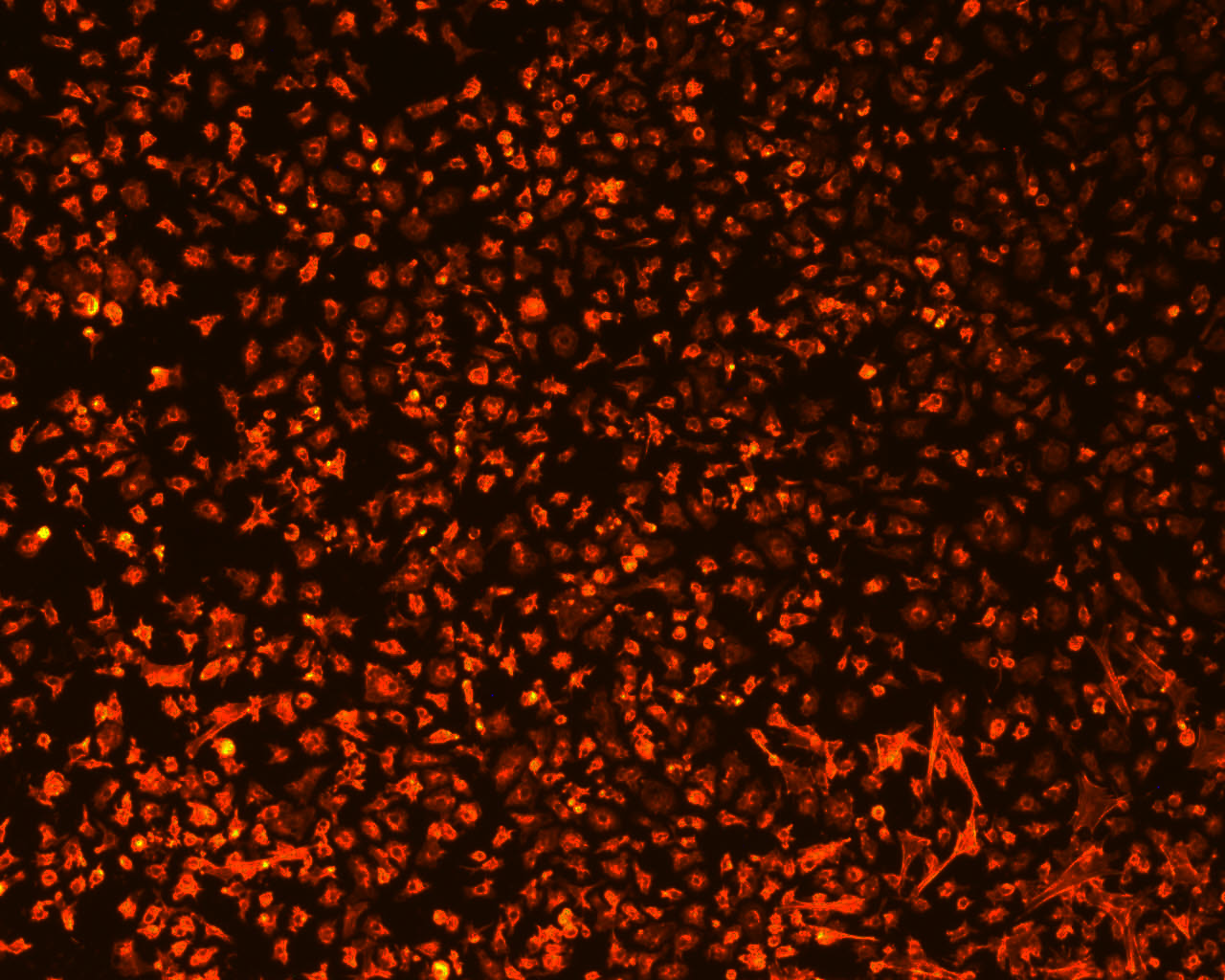

Supplement: Data S1 [file peerj-09-10898-s003.zip › Figure3E-MCSF.tif]

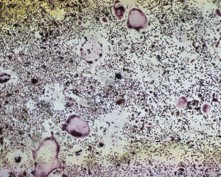

Supplement: Data S1 [file peerj-09-10898-s003.zip › Figure4C-MCSF+RANKL-D0+ucOCN.jpg]

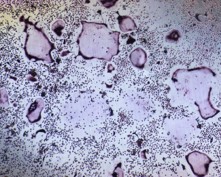

Supplement: Data S1 [file peerj-09-10898-s003.zip › Figure4C-MCSF+RANKL-D2+ucOCN.jpg]

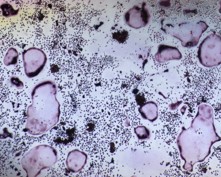

Supplement: Data S1 [file peerj-09-10898-s003.zip › Figure4C-MCSF+RANKL-D4+ucOCN.jpg]

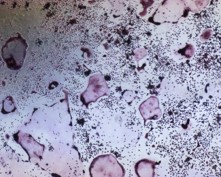

Supplement: Data S1 [file peerj-09-10898-s003.zip › Figure4C-MCSF+RANKL.jpg]

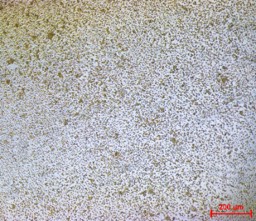

Supplement: Data S1 [file peerj-09-10898-s003.zip › Figure4C-MCSF.jpg]

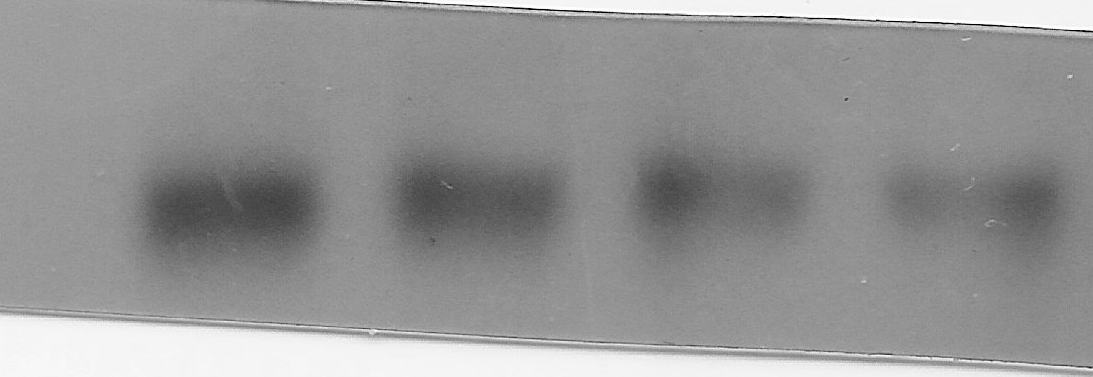

Supplement: Data S1 [file peerj-09-10898-s003.zip › Figure4E-c-fms.jpg]

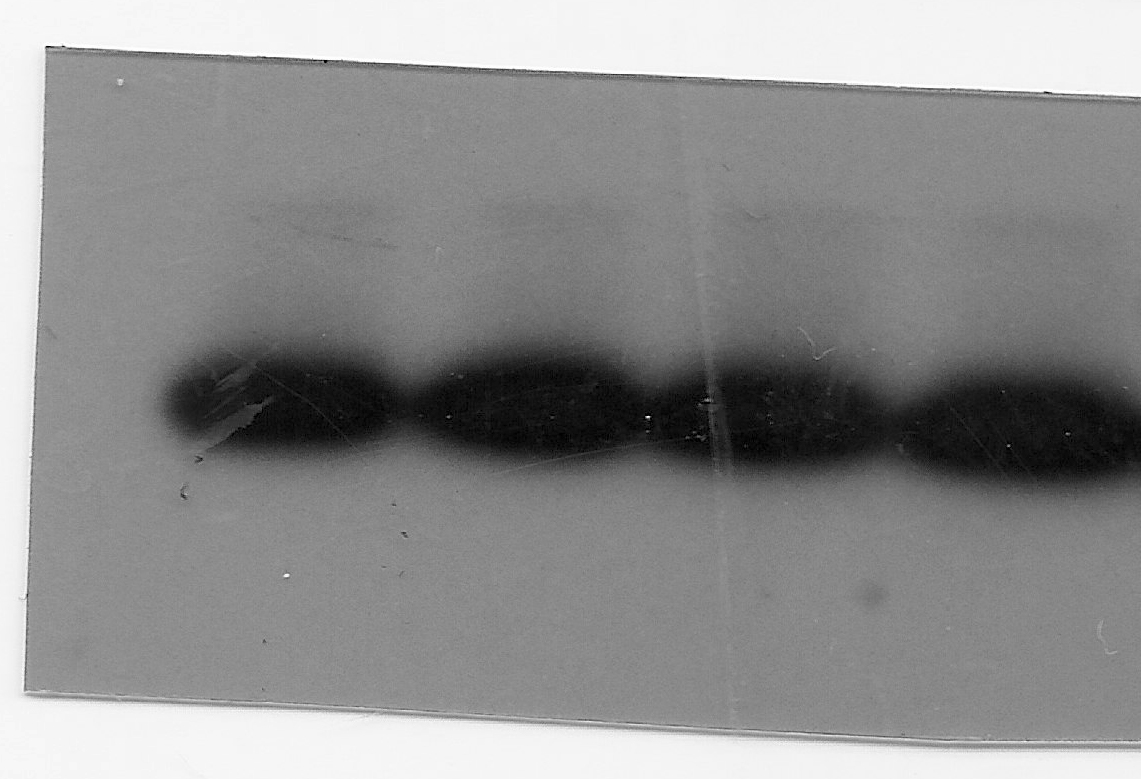

Supplement: Data S1 [file peerj-09-10898-s003.zip › Figure4E-GAPDH.jpg]

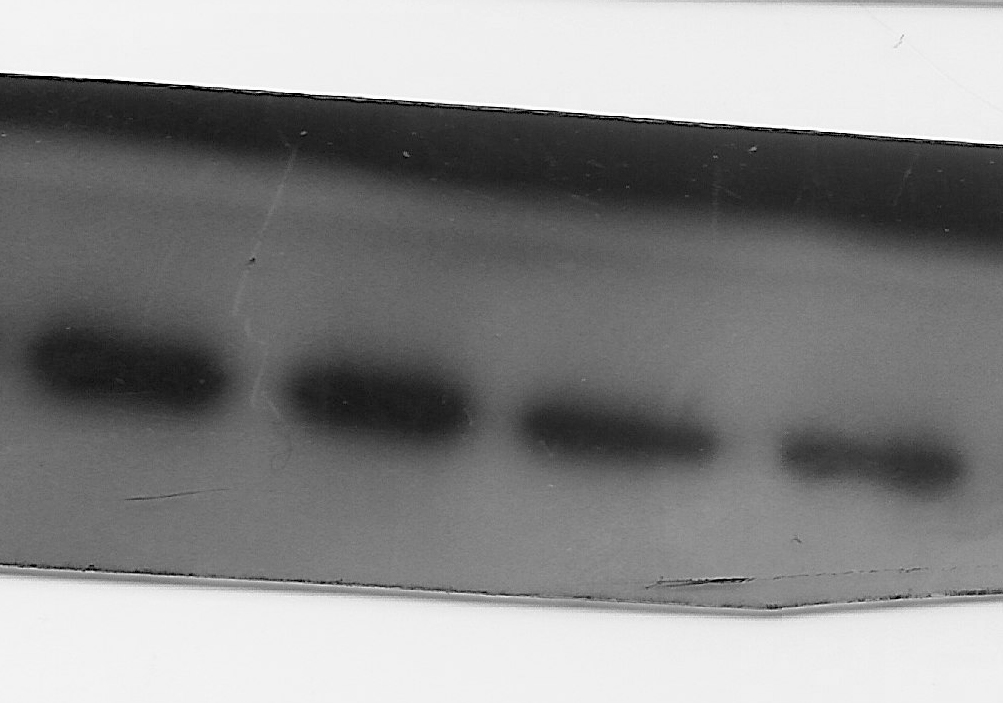

Supplement: Data S1 [file peerj-09-10898-s003.zip › Figure4E-Rank.jpg]

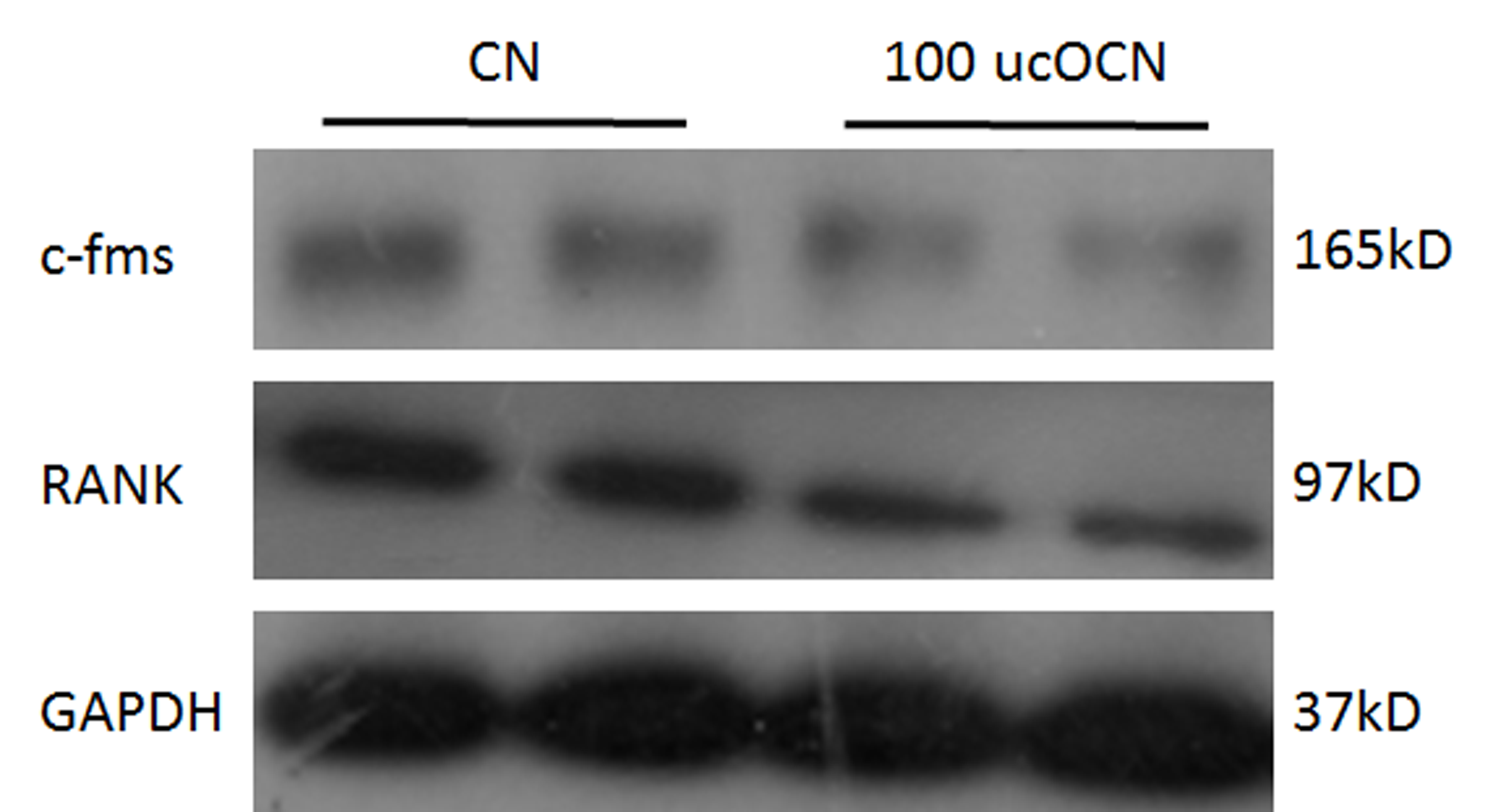

Supplement: Data S1 [file peerj-09-10898-s003.zip › Figure4E.tif]

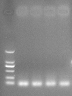

Supplement: Data S1 [file peerj-09-10898-s003.zip › Figure5A-GAPDH.jpg]

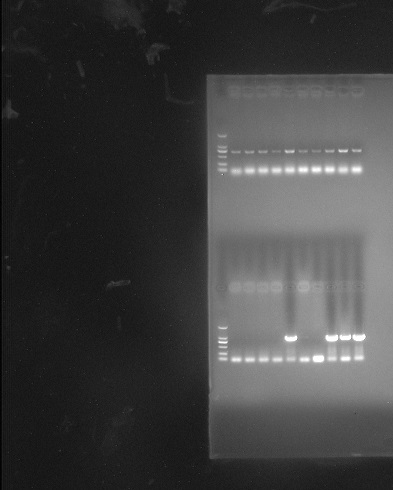

Supplement: Data S1 [file peerj-09-10898-s003.zip › Figure5A-Gprc6a+GAPDH.jpg]

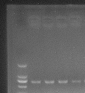

Supplement: Data S1 [file peerj-09-10898-s003.zip › Figure5A-Gprc6a.jpg]

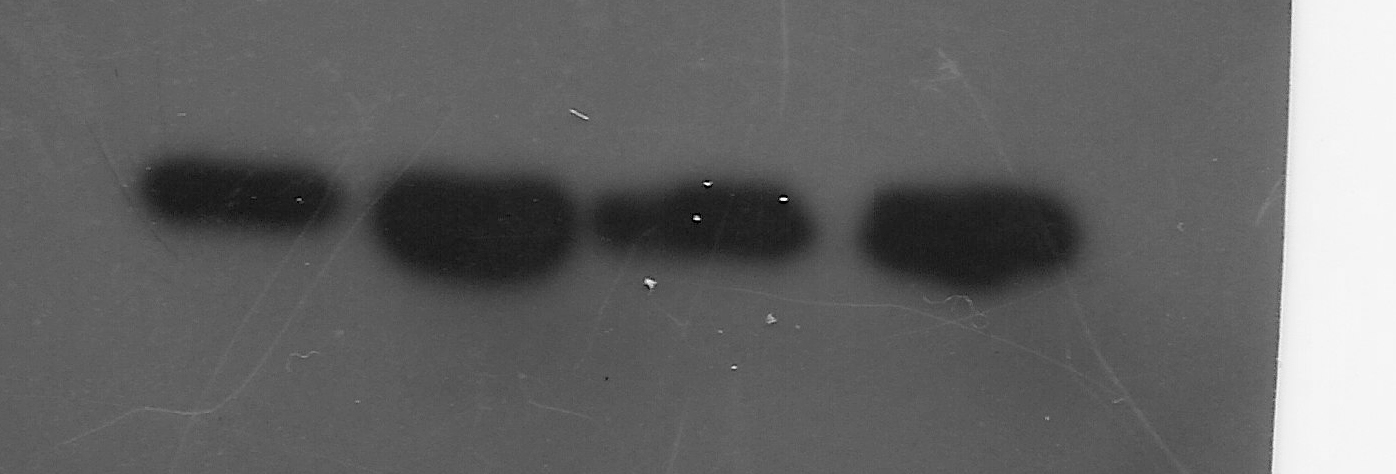

Supplement: Data S1 [file peerj-09-10898-s003.zip › Figure5B-GAPDH.jpg]

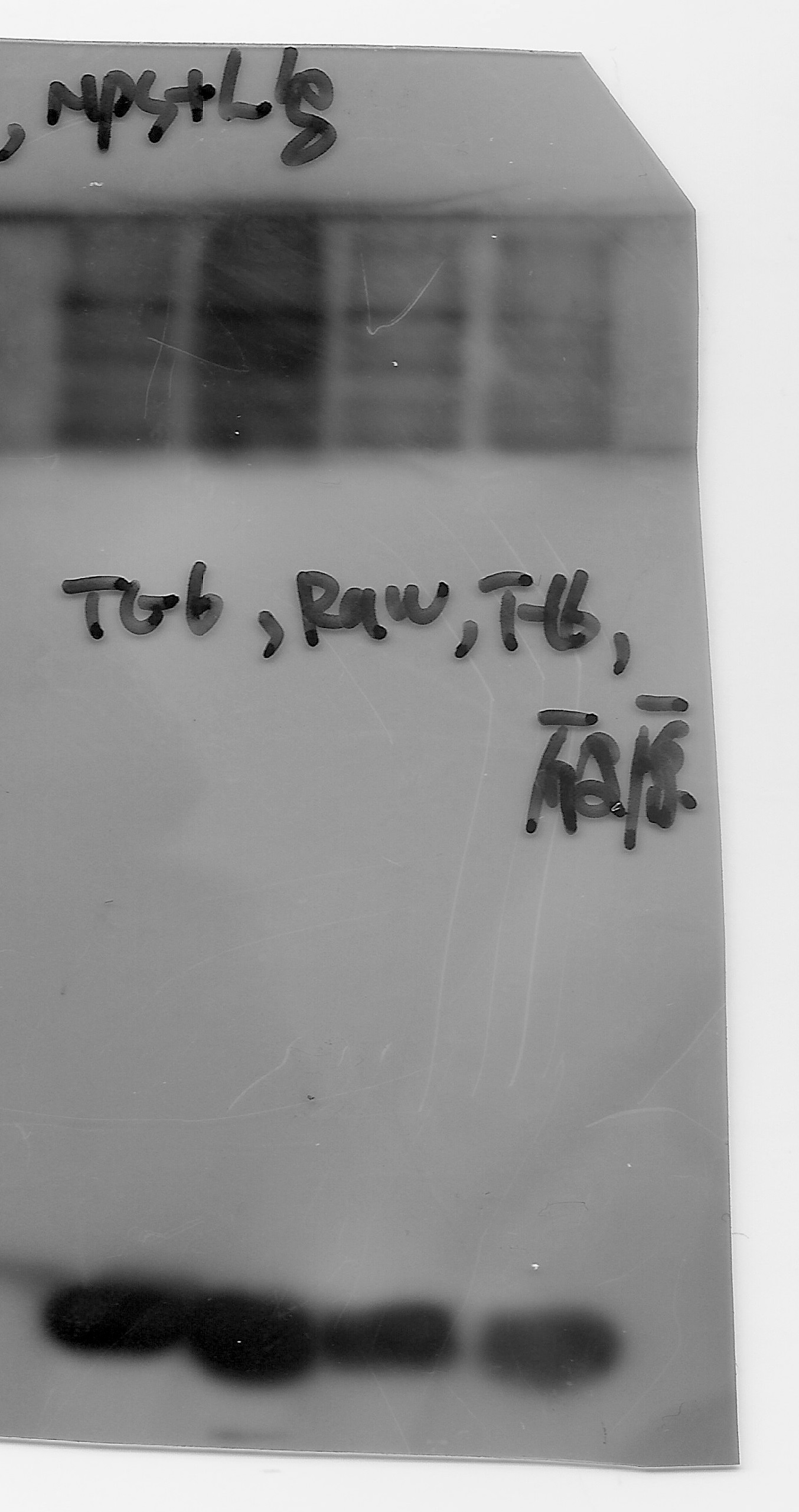

Supplement: Data S1 [file peerj-09-10898-s003.zip › Figure5B-Gprc6a+GAPDH.jpg]

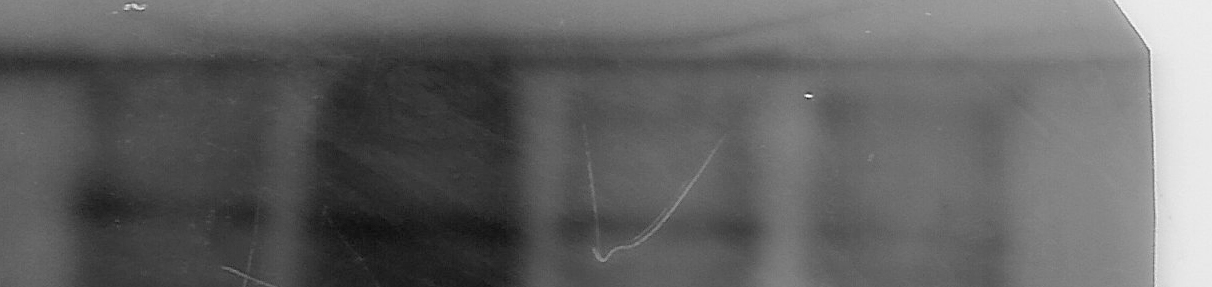

Supplement: Data S1 [file peerj-09-10898-s003.zip › Figure5B-Gprc6a.jpg]

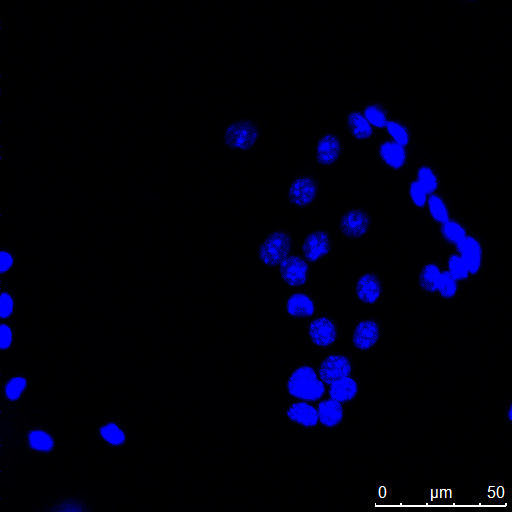

Supplement: Data S1 [file peerj-09-10898-s003.zip › Figure5C-BMM-DAPI.tif]

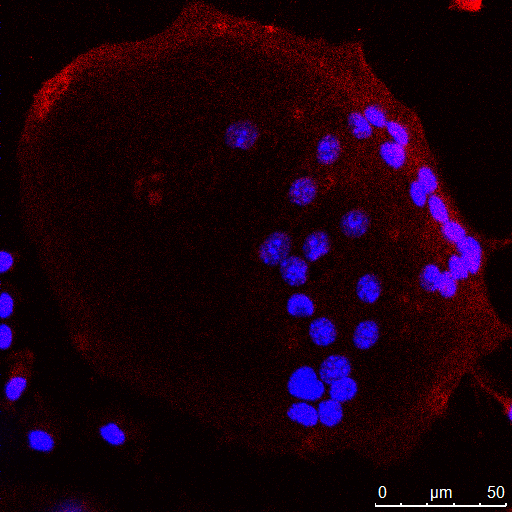

Supplement: Data S1 [file peerj-09-10898-s003.zip › Figure5C-BMM-merged.tif]

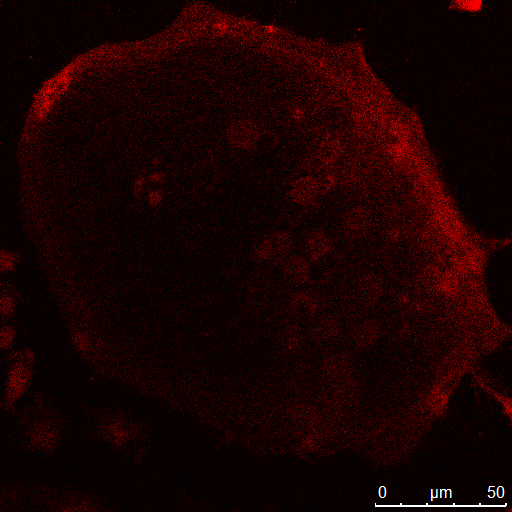

Supplement: Data S1 [file peerj-09-10898-s003.zip › Figure5C-BMM-TRITC.tif]

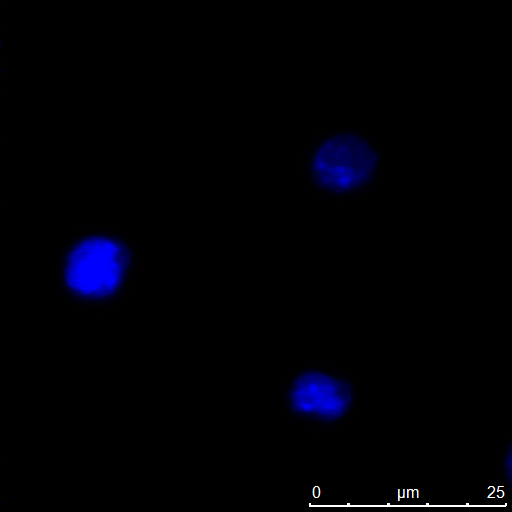

Supplement: Data S1 [file peerj-09-10898-s003.zip › Figure5C-Raw264.7-DAPI.tif]

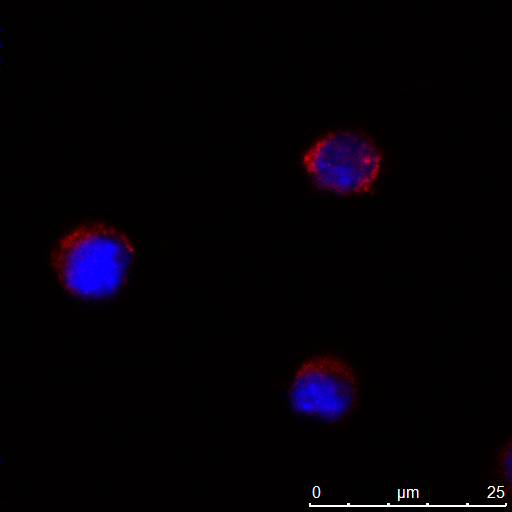

Supplement: Data S1 [file peerj-09-10898-s003.zip › Figure5C-Raw264.7-merged.tif]

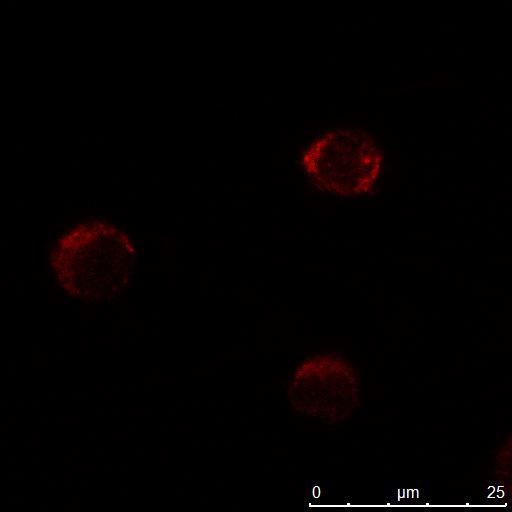

Supplement: Data S1 [file peerj-09-10898-s003.zip › Figure5C-Raw264.7-TRITC.tif]

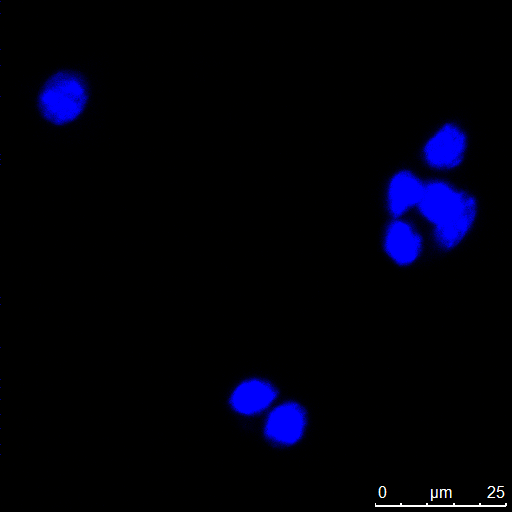

Supplement: Data S1 [file peerj-09-10898-s003.zip › Figure5C-TC6-2-DAPI.tif]

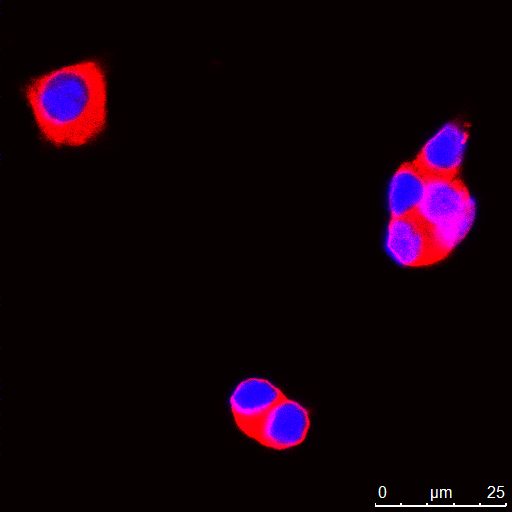

Supplement: Data S1 [file peerj-09-10898-s003.zip › Figure5C-TC6-2-merged.tif]

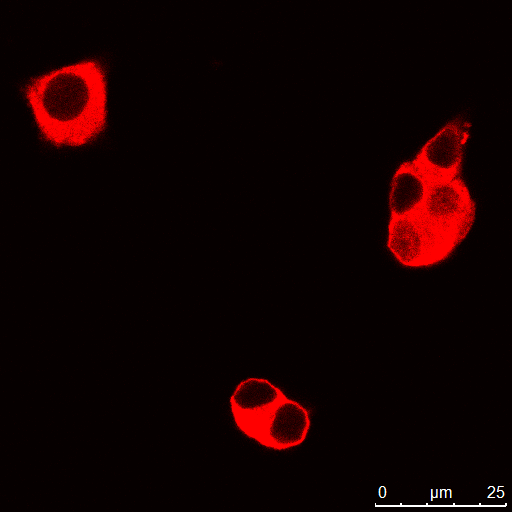

Supplement: Data S1 [file peerj-09-10898-s003.zip › Figure5C-TC6-2-TRITC.tif]

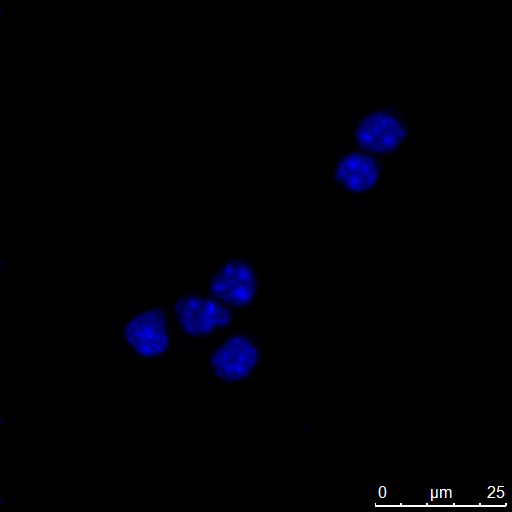

Supplement: Data S1 [file peerj-09-10898-s003.zip › Figure5C-TC6-DAPI.tif]

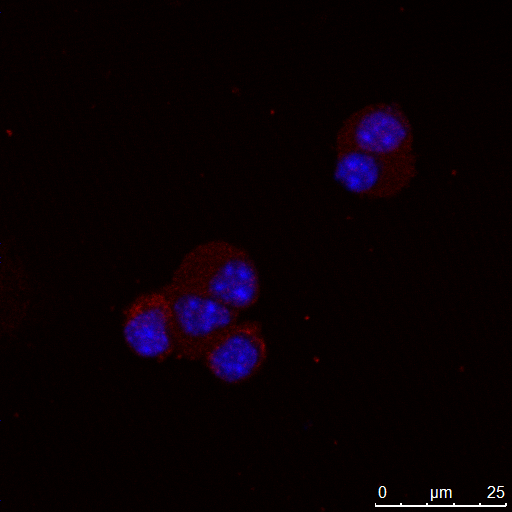

Supplement: Data S1 [file peerj-09-10898-s003.zip › Figure5C-TC6-merged-DAPI.tif]

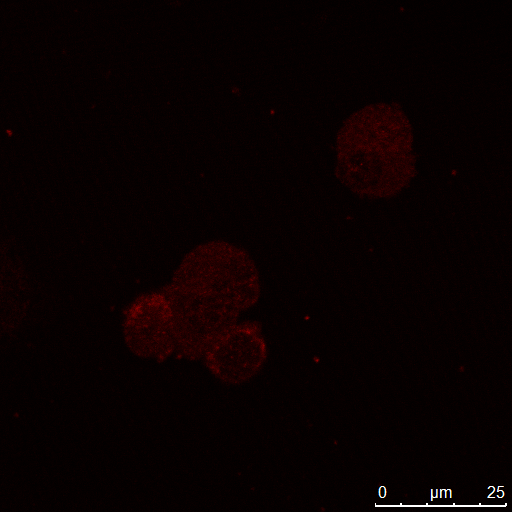

Supplement: Data S1 [file peerj-09-10898-s003.zip › Figure5C-TC6-TRITC.tif]

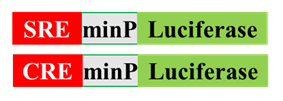

Supplement: Data S1 [file peerj-09-10898-s003.zip › Figure5D.jpg]

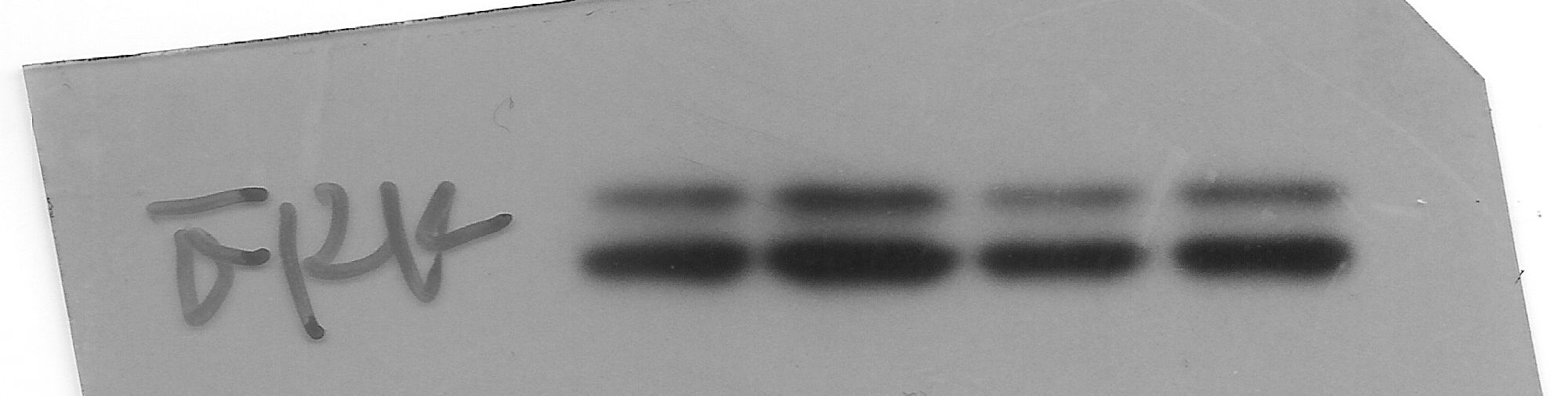

Supplement: Data S1 [file peerj-09-10898-s003.zip › Figure5I-ERK.jpg]

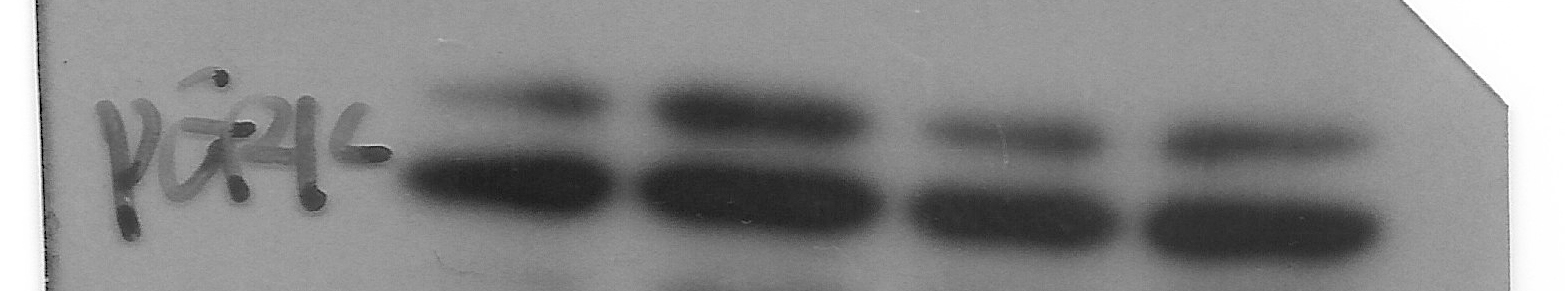

Supplement: Data S1 [file peerj-09-10898-s003.zip › Figure5I-pERK.jpg]
